# Maintenance of graft tissue–resident Foxp3^+^ cells is necessary for lung transplant tolerance in mice

**DOI:** 10.1172/JCI178975

**Published:** 2025-03-18

**Authors:** Wenjun Li, Yuriko Terada, Yun Zhu Bai, Yuhei Yokoyama, Hailey M. Shepherd, Junedh M. Amrute, Amit I. Bery, Zhiyi Liu, Jason M. Gauthier, Marina Terekhova, Ankit Bharat, Jon H. Ritter, Varun Puri, Ramsey R. Hachem, Hēth R. Turnquist, Peter T. Sage, Alessandro Alessandrini, Maxim N. Artyomov, Kory J. Lavine, Ruben G. Nava, Alexander S. Krupnick, Andrew E. Gelman, Daniel Kreisel

**Affiliations:** 1 Department of Surgery,; 2 Department of Medicine, and; 3 Department of Pathology and Immunology, Washington University in St. Louis, St. Louis, Missouri, USA.; 4 Department of Surgery, Northwestern University, Chicago, Illinois, USA.; 5 Department of Surgery, Starzl Transplantation Institute, University of Pittsburgh, Pittsburgh, Pennsylvania, USA.; 6 Transplantation Research Center, Renal Division, Brigham and Women’s Hospital, Harvard Medical School, Boston, Massachusetts, USA.; 7 Department of Surgery, Center for Transplantation Sciences, Massachusetts General Hospital, Boston, Massachusetts, USA.; 8 Department of Surgery, University of Maryland, Baltimore, Maryland, USA.

**Keywords:** Immunology, Transplantation, Tolerance

## Abstract

Mechanisms that mediate allograft tolerance differ between organs. We have previously shown that Foxp3^+^ T cell–enriched bronchus-associated lymphoid tissue (BALT) is induced in tolerant murine lung allografts and that these Foxp3^+^ cells suppress alloimmune responses locally and systemically. Here, we demonstrated that Foxp3^+^ cells that reside in tolerant lung allografts differed phenotypically and transcriptionally from those in the periphery and were clonally expanded. Using a mouse lung retransplant model, we showed that recipient Foxp3^+^ cells were continuously recruited to the BALT within tolerant allografts. We identified distinguishing features of graft-resident and newly recruited Foxp3^+^ cells and showed that graft-infiltrating Foxp3^+^ cells acquired transcriptional profiles resembling those of graft-resident Foxp3^+^ cells over time. Allografts underwent combined antibody-mediated rejection and acute cellular rejection when recruitment of recipient Foxp3^+^ cells was prevented. Finally, we showed that local administration of IL-33 could expand and activate allograft-resident Foxp3^+^ cells, providing a platform for the design of tolerogenic therapies for lung transplant recipients. Our findings establish graft-resident Foxp3^+^ cells as critical orchestrators of lung transplant tolerance and highlight the need to develop lung-specific immunosuppression.

## Abstract 2

Lung transplant tolerance is critically dependent upon the maintenance of a graft-resident immunoregulatory cell population

## Graphical abstract

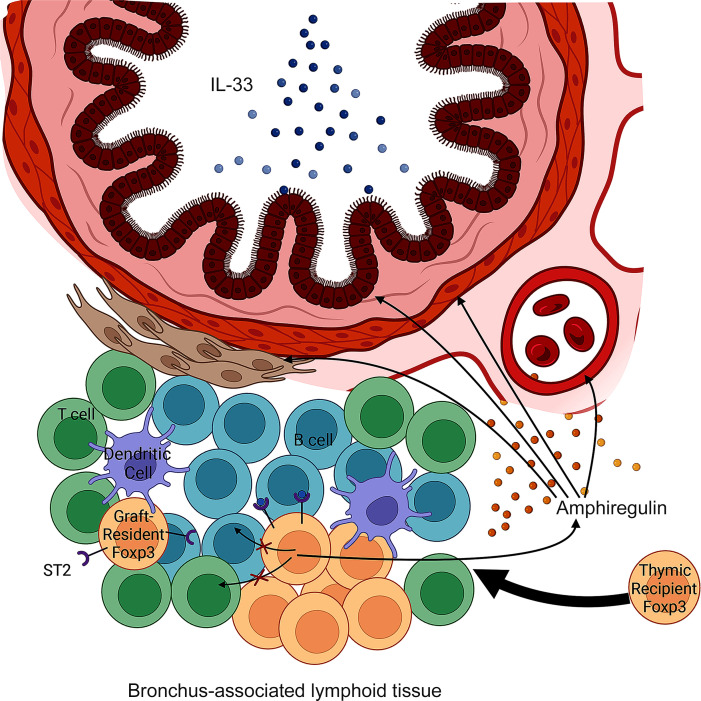

## Introduction

Lung transplantation remains the only treatment option for many patients afflicted with end-stage pulmonary disease. However, outcomes after lung transplantation are poor in comparison with other solid organ transplants, with 5-year survival of about 60% (1). This is largely due to the substantial proportion of patients who develop chronic lung allograft dysfunction, which is a manifestation of chronic rejection. We have suggested that this high rate of graft failure is due to the clinical use of immunosuppressive agents that do not account for the unique immunological properties of lungs (2). For example, our group has described that several cell populations (e.g., memory CD8^+^ T cells, eosinophils) that can trigger rejection after the transplantation of other organs may be critical for the downregulation of alloimmune responses after lung transplantation (3–5). Furthermore, while immune cell interactions in draining lymph nodes regulate graft acceptance after heart and pancreatic islet transplantation, we have shown that immune circuits that maintain tolerance after lung transplantation are established within the graft itself (6–9). Therefore, as immune pathways that regulate tolerance are not universal but rather organ specific, a better understanding of mechanisms that mediate tolerance after lung transplantation represents an unmet need.

Our group has previously demonstrated that bronchus-associated lymphoid tissue (BALT), a tertiary lymphoid organ, is induced in tolerant lung allografts (7). Using a mouse lung retransplant model, we have shown that Foxp3^+^ cells that accumulate within the BALT of tolerant lung allografts prevent the local activation of humoral immunity (6). Thus, graft tissue–resident Foxp3^+^ cells play a central role in lung transplant tolerance. Furthermore, pathogens that are known risk factors for lung allograft rejection can hinder the suppressive functions of Foxp3^+^ cells, raising the possibility that environmental stresses can disrupt tolerance after pulmonary transplantation (10). Therefore, it is important to define mechanisms that maintain a stable population of Foxp3^+^ cells within lung grafts.

In this study, we demonstrate that lung graft–resident Foxp3^+^ cells are phenotypically and transcriptionally distinct from Foxp3^+^ cells in the periphery of tolerant mice. Graft-resident Foxp3^+^ cells require ongoing replenishment by recipient-derived thymic Foxp3^+^ cells to maintain tolerance. Preventing the continuous recruitment of Foxp3^+^ cells triggers graft rejection with histological features of antibody-mediated rejection (AMR) and acute cellular rejection (ACR). Finally, we explore a potential therapeutic approach to expand the graft-resident Foxp3^+^ cell population through local cytokine delivery.

## Results

### Allograft-resident and spleen Foxp3^+^ cells are phenotypically and transcriptionally distinct in tolerant lung transplant recipients.

**Figure 1:**
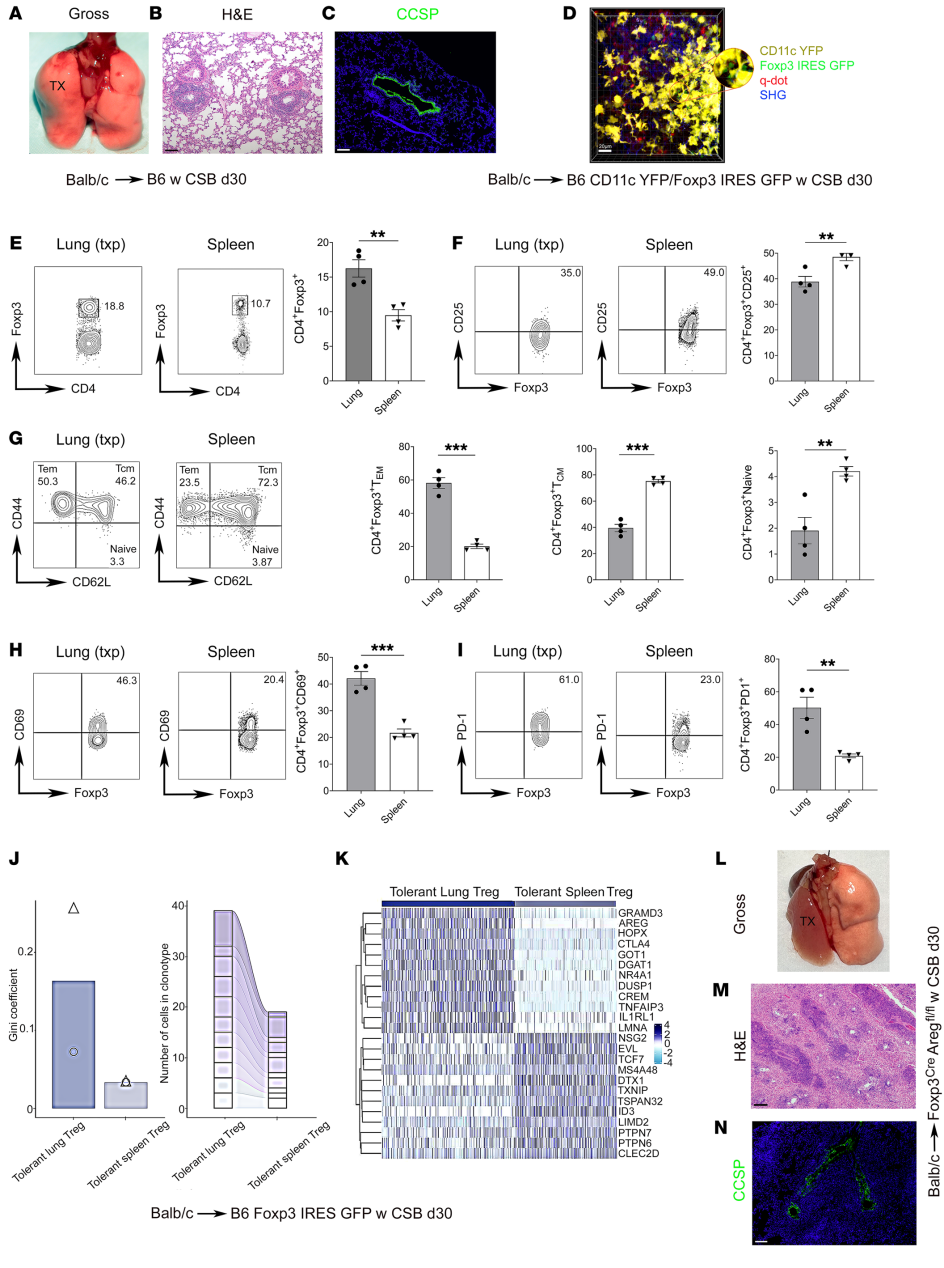
Allograft-resident and spleen Foxp3^+^ cells are phenotypically and transcriptionally distinct in tolerant lung transplant recipients. (**A**–**C**) Gross image (*n* = 6) (**A**), H&E staining (*n* = 6) (**B**), and CCSP immunofluorescence staining (*n* = 2) (**C**) of left lung transplant from BALB/c donor into CSB-treated B6 recipient, at least 30 days after engraftment. Scale bars: 100 μm. (**D**) Two-photon intravital microscopy of BALB/c lung at least 30 days after transplantation into CSB-treated B6 CD11c-EYFP/Foxp3-GFP recipient (*n* = 3). CD11c^+^ cells are yellow, Foxp3^+^ cells are green, collagen appears blue owing to second-harmonic generation (SHG), and vessels are red following i.v. injection of quantum dots. Scale bar: 20 μm. (**E**–**I**) Representative flow cytometry plots and quantification of abundance of Foxp3-expressing CD45^+^CD90.2^+^CD4^+^CD8^–^ T cells (**E**), CD25-expressing CD45^+^CD90.2^+^CD4^+^CD8^–^Foxp3^+^ T cells (CD25 expression determined based on isotype control staining) (**F**), effector memory (CD44^hi^CD62L^lo^), central memory (CD44^hi^CD62L^hi^), and naive (CD44^lo^CD62L^hi^) CD45^+^CD90.2^+^CD4^+^CD8^–^Foxp3^+^ T cells (**G**), CD69-expressing CD45^+^CD90.2^+^CD4^+^CD8^–^Foxp3^+^ T cells (**H**), and (**I**) PD-1–expressing CD45^+^CD90.2^+^CD4^+^CD8^–^Foxp3^+^ T cells in BALB/c lung and recipient spleen at least 30 days after transplantation into CSB-treated B6 recipients (*n* = 4). At least 30 days after transplantation of BALB/c lungs into CSB-treated B6 Foxp3-GFP mice, CD45^+^CD90.2^+^CD4^+^CD8^–^GFP^+^ cells were sorted and processed for TCR and genome sequencing (*n* = 2). (**J**) Gini coefficient (0 represents maximal diversity) comparison of clonal expansion (left) and number of cells in the top 11 shared clonotypes between Foxp3^+^ cells in tolerant lung allografts and recipient spleens (right) (triangles and circles denote individual mice). (**K**) Heatmap of most highly differentially expressed genes in Foxp3^+^ cells between tolerant lung allografts and recipient spleens (2 pooled lungs and spleens). (**L**–**N**) Gross image (*n* = 4) (**L**), H&E staining (*n* = 4) (**M**), and CCSP immunofluorescence staining (*n* = 2) (**N**) of BALB/c lungs 30 days after transplantation into CSB-treated B6 Foxp3-YFP-Cre *Areg^fl/fl^* mice. Scale bars: 100 μm. Results expressed as mean ± SEM. **P < 0.01, ***P < 0.001. d30, day 30; q-dot, quantum dot; Tcm, T central memory; Tem, T effector memory; Treg, regulatory Foxp3^+^ T cell; TX, transplanted lung.

BALT develops in tolerant BALB/c lung allografts by 30 days after transplantation into C57BL/6 (B6) recipients that receive perioperative costimulatory blockade (CSB) (Figure 1, A and B) (7). The allografts had virtually no evidence of inflammation or cellular rejection, and expression of club cell secretory protein (CCSP) was preserved in the airway epithelium of these grafts (Figure 1C and Supplemental Figure 1; supplemental material available online with this article; https://doi.org/10.1172/JCI178975DS1). We next performed allogeneic transplants from BALB/c donors into CSB-treated B6 CD11c–enhanced yellow fluorescent protein (EYFP)/Foxp3–green fluorescent protein (GFP) recipients followed by 2-photon intravital imaging 30 days after engraftment (11). We noted that recipient Foxp3^+^ cells were predominantly located in close proximity to clusters of recipient CD11c^+^ antigen-presenting cells, which we and others have previously reported to reside in T cell zones of BALT (Figure 1D) (7, 12, 13). We then compared Foxp3^+^ cells that resided in tolerant lung allografts with Foxp3^+^ cells in the spleens of tolerant mice. We noted a higher abundance of Foxp3-expressing CD4^+^ T cells in lung allografts (Figure 1E). In addition, we observed lower levels of CD25 expression in graft-resident Foxp3^+^ cells, a shift toward an effector memory phenotype, and higher expression levels of CD69 and PD-1 in comparison with Foxp3 cells in the spleen (Figure 1, F–I).

To investigate the gene expression patterns of Foxp3^+^ cells in lung grafts compared with those in the spleen, we next transplanted BALB/c lungs into B6 Foxp3-GFP recipients that were treated with perioperative CSB. Thirty days later, CD4^+^GFP^+^ cells were sorted from lung allografts and spleens and subjected to single-cell RNA sequencing. From these data, we synthesized an integrated dataset consisting of 946 and 727 tolerant lung and spleen Foxp3^+^ cells, respectively. We examined the single-cell T cell receptor (TCR) profiles of graft- and spleen-resident Foxp3^+^ cells. Foxp3^+^ cells within grafts were more clonally expanded than those in the spleen (Figure 1J, left). While particular clonotypes were shared between both tissues, the most expanded ones were unique for the lung allograft tissue (Figure 1J, right). Differential gene analysis revealed markedly altered gene expression profiles in Foxp3^+^ cells in tolerant lungs compared with those isolated from spleens of tolerant recipients (Figure 1K). Notably, *Areg* (encoding amphiregulin), *Ctla4*, and *Il1r1* (encoding ST2, the receptor for IL-33) were among the most highly differentially expressed genes in graft-resident Foxp3^+^ cells. To examine whether amphiregulin expression in Foxp3^+^ cells is functionally important, we next evaluated BALB/c lungs 30 days after transplantation into B6 recipients that lack *Areg* expression in Foxp3^+^ cells (Foxp3-YFP-Cre *Areg^fl/fl^*). Compared with control conditions, these allografts were not ventilated and had neutrophilic capillaritis and alveolar inflammation, characteristic features of AMR, and reduced expression of CCSP along with histological evidence of severe ACR (Figure 1, L–N, and Supplemental Figure 2). Amphiregulin is a ligand for the epidermal growth factor receptor (EGFR). To gain mechanistic insight into how Foxp3^+^ cell–derived amphiregulin prevents allograft rejection, we transplanted BALB/c lungs into CSB-treated B6 Foxp3-YFP-Cre *Areg^fl/fl^* mice or B6 Foxp3-YFP-Cre controls and examined allografts by single-nuclear RNA sequencing 14 days later (Supplemental Figure 3A). We found that EGFR expression in the grafts was mostly restricted to stromal cells, including mesothelium, fibroblasts, smooth muscle cells, and type II alveolar epithelium (Supplemental Figure 3B). Mesothelial cells harbored the largest number of differentially expressed genes between B6 Foxp3-YFP-Cre *Areg^fl/fl^* recipients and controls (Supplemental Figure 3C). We identified 2 subtypes or states of mesothelial cells with a relative enrichment of the subtype that expresses *Col5a1*, *Col6a3*, *Col4a6*, and *Lum* after transplantation into mice that lack *Areg* in Foxp3^+^ cells (Supplemental Figure 3, D–F). We next examined transbronchial biopsies from human lung transplant patients, who had BALT with no evidence of ACR (grade A0). In 4 of 5 such biopsies we observed colocalization of staining for Foxp3 and amphiregulin (Supplemental Figure 4).

### Graft-infiltrating Foxp3^+^ cells are derived from the thymus rather than from peripheral conversion of Foxp3^–^ T cells.

**Figure 2:**
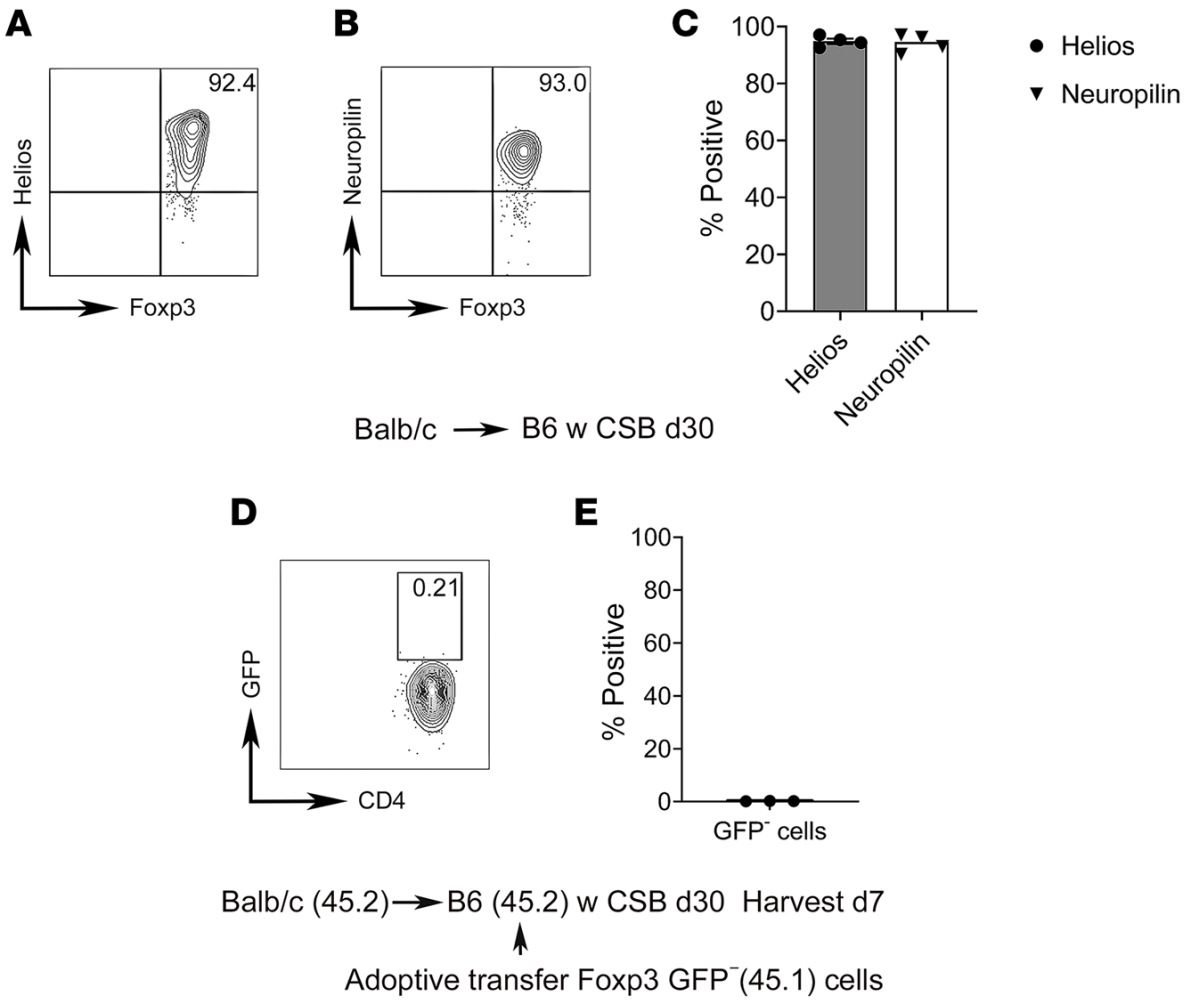
Graft-infiltrating Foxp3^+^ cells are derived from the thymus rather than from peripheral conversion of Foxp3^–^ T cells. (**A**–**C**) Representative flow cytometric plots (**A** and **B**) and quantification (**C**) of Helios and neuropilin-1 expression by CD45^+^CD90.2^+^CD4^+^CD8^–^Foxp3^+^ T cells in BALB/c lungs at least 30 days after transplantation into CSB-treated B6 recipients (*n* = 4). Thirty days after transplantation of BALB/c (CD45.2) lungs into CSB-treated B6 (CD45.2) recipients, CD90.2^+^CD4^+^GFP^–^ T cells, isolated from secondary lymphoid organs of B6 Foxp3-GFP (CD45.1) reporter mice, were injected i.v. Lungs were analyzed 7 days later. (**D** and **E**) Representative flow cytometric plot and analysis of GFP expression in adoptively transferred cells (CD45.1^+^) in lung allografts (*n* = 3). Results expressed as mean ± SEM.

We next examined the expression of Helios and neuropilin-1 on Foxp3^+^ cells that reside in tolerant lung allografts at least 30 days after lung transplantation. These markers have been previously associated with thymic origin of regulatory T cells (14–16). Over 90% of graft-resident Foxp3^+^ cells expressed these markers (Figure 2, A–C). To assess whether non-regulatory T cells convert into regulatory T cells within tolerant pulmonary allografts, we transplanted BALB/c (CD45.2) lungs into B6 (CD45.2) hosts that received perioperative CSB. At least 30 days later we injected CD90^+^CD4^+^GFP^–^ T cells into these recipients that were isolated from spleen and lymph nodes of congenic (CD45.1) B6 Foxp3-GFP reporter mice. While we were able to detect adoptively transferred CD45.1^+^CD90^+^CD4^+^ T cells in tolerant lung allografts 7 days after injection, they expressed virtually no GFP (Figure 2, D and E). Collectively, these data indicate that Foxp3^+^ cells within tolerant lung allografts are derived from the thymus rather than from peripheral conversion of non-regulatory T cells.

### Newly recruited Foxp3^+^ cells infiltrate BALT within tolerant lung allografts and differ transcriptionally from graft-resident Foxp3^+^ cells.

**Figure 3:**
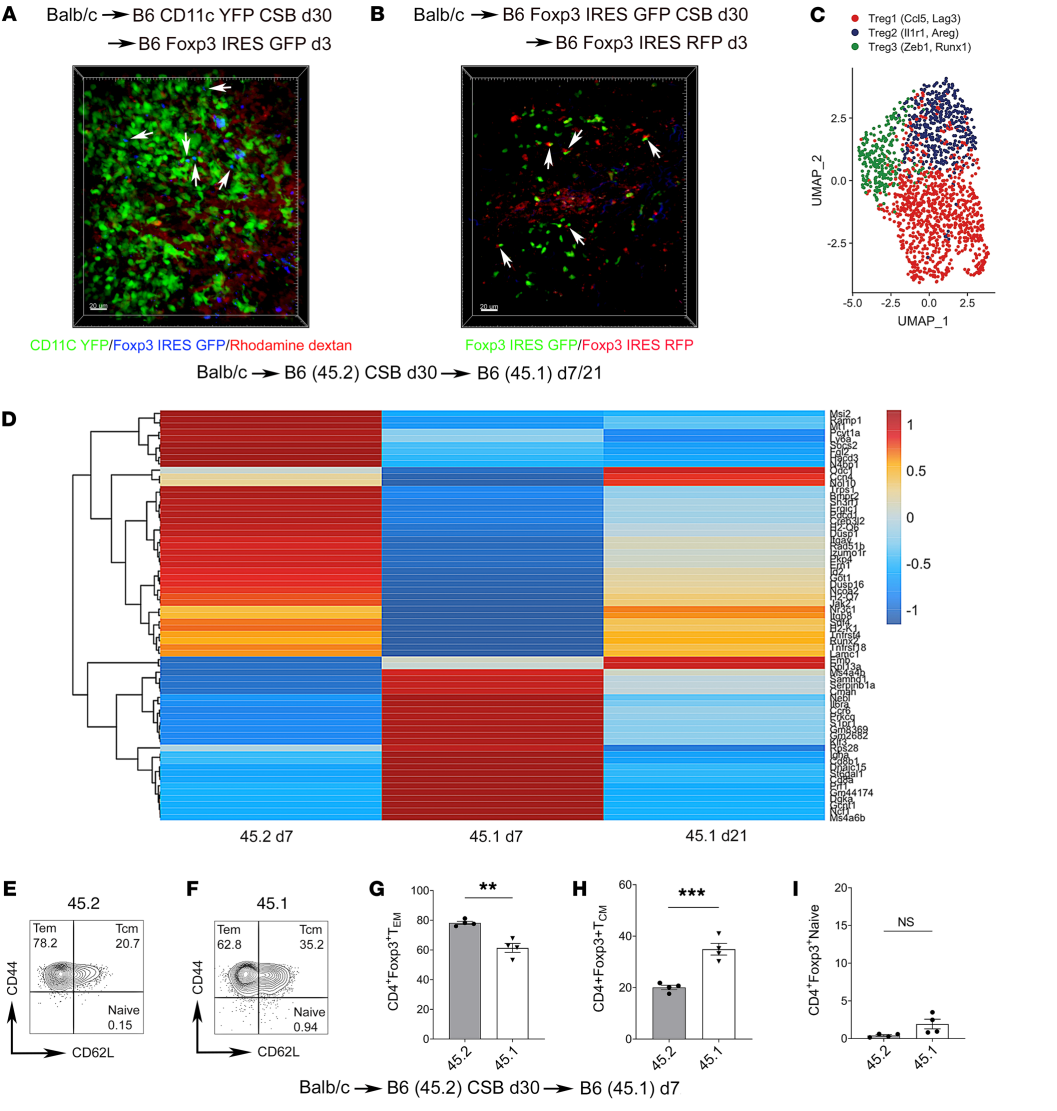
Newly recruited Foxp3^+^ cells infiltrate BALT within tolerant lung allografts and differ transcriptionally from graft-resident Foxp3^+^ cells. (**A**) BALB/c lungs initially transplanted into CSB-treated B6 CD11c-EYFP mice and then at least 30 days later retransplanted into non-immunosuppressed B6 Foxp3-GFP hosts were imaged with intravital 2-photon microscopy 3 days after retransplantation (*n* = 3). White arrows point to contacts between Foxp3^+^ (blue) and CD11c^+^ (green) cells within the BALT of lung allografts. Rhodamine-dextran labels vessels red. (**B**) BALB/c lungs initially transplanted into CSB-treated B6 Foxp3-GFP mice and then at least 30 days later retransplanted into non-immunosuppressed B6 Foxp3-RFP recipients were imaged with intravital 2-photon microscopy 3 days after retransplantation (*n* = 3). White arrows point to contacts between graft-resident (green) and graft-infiltrating (red) Foxp3^+^ cells within lung allografts. (**C** and **D**) BALB/c (CD45.2) lungs were transplanted into CSB-treated B6 (CD45.2) recipients and at least 30 days later retransplanted into non-immunosuppressed B6 (CD45.1) mice. Seven and 21 days after retransplantation, graft-resident (CD45.2) (7 days) and extravasated graft-infiltrating (CD45.1) (7 and 21 days) T cells were sorted from the lung allografts (samples were collected from 4 retransplant recipients and pooled) and processed for single-cell RNA sequencing. (**C**) Uniform manifold approximation and projection (UMAP) embedding plot of regulatory T cell subpopulations. (**D**) Heatmap of statistically significant (log_2_ fold change > 0.25, adjusted *P* value < 0.05) differentially expressed genes between graft-resident CD45.2 (day 7) and extravasated graft-infiltrating CD45.1 (days 7 and 21) regulatory T cells grouped by condition. (**E**–**I**) Representative flow cytometry plots and quantification of abundance of effector memory (CD44^hi^CD62L^lo^), central memory (CD44^hi^CD62L^hi^), and naive (CD44^lo^CD62L^hi^) graft-resident CD45.2^+^ versus graft-infiltrating CD45.1^+^CD90.2^+^CD4^+^CD8^–^Foxp3^+^ T cells on day 7 (*n* = 4). Results expressed as mean ± SEM. Scale bars: 20 μm. **P < 0.01, ***P < 0.001. RFP, red fluorescent protein; Tcm, T central memory; Tem, T effector memory.

We previously reported that tolerant lung allografts maintain a tolerant state after being retransplanted into non-immunosuppressed recipients (6, 7, 17). Importantly, the retransplant model allows us to distinguish between graft-resident and graft-infiltrating cells using congenic or fluorescent markers. To evaluate recruitment patterns of Foxp3^+^ cells that infiltrate tolerant lung allografts, we transplanted BALB/c lungs into B6 CD11c-EYFP recipients that were treated with perioperative CSB. At least 30 days later we retransplanted these lung allografts into non-immunosuppressed B6 Foxp3-GFP secondary recipients and performed intravital microscopy 3 days later. We found that recipient-derived graft-infiltrating Foxp3^+^ cells predominantly infiltrated BALT, hallmarked by CD11c^+^ cell aggregates, where they made close contact with these CD11c^+^ cells (Figure 3A and Supplemental Video 1). Moreover, in retransplants of BALB/c lungs with CSB-treated B6 Foxp3-IRES-GFP primary recipients and non-immunosuppressed B6 Foxp3–IRES–red fluorescent protein (RFP) secondary recipients, we observed that graft-infiltrating Foxp3^+^ (RFP) cells also interacted with graft-resident Foxp3^+^ (GFP) cells (Figure 3B and Supplemental Video 2).

To compare gene expression profiles of graft-resident and newly recruited Foxp3^+^ cells, we transplanted BALB/c (CD45.2) lungs into B6 (CD45.2) mice that received perioperative CSB and retransplanted these allografts into non-immunosuppressed B6 (CD45.1) recipients at least 30 days later. Seven and 21 days after retransplantation, we obtained single-cell RNA sequencing data of sorted CD45.2^+^ and CD45.1^+^ cells from lung allografts. We injected a fluorochrome-labeled anti-CD45.1 antibody i.v. 5 minutes before sacrifice to exclude intravascular recipient CD45.1^+^ cells from the analysis. We identified 8 T cell populations (Supplemental Figure 5, A and B). We evaluated Foxp3 cells within this dataset, which had 3 discrete subpopulations (Figure 3C and Supplemental Figure 6A). We found that graft-resident (CD45.2^+^) regulatory T cells exhibited a markedly distinct transcriptional profile compared with graft-infiltrating (CD45.1^+^) extravasated regulatory T cells 7 days after retransplantation (Figure 3D). Graft-infiltrating (CD45.1^+^) extravasated regulatory T cells shifted their transcriptional profile at 21 compared with 7 days after retransplantation. The transcriptional profile of day 21 graft-infiltrating (CD45.1^+^) extravasated regulatory T cells revealed downregulation of many genes expressed in these cells at day 7 after retransplantation and began to resemble the transcriptional profile of graft-resident (CD45.2^+^) regulatory T cells at 7 days after retransplantation. Most notably, day 7 graft-resident (CD45.2^+^) and day 21 graft-infiltrating (CD45.1^+^) extravasated regulatory T cells had higher expression of *Areg* (encoding amphiregulin), *Ctla4* (encoding CTLA4), *Tnfrsf18* (encoding CD357/glucocorticoid-induced TNFR-related protein [GITR]), *Tnfaip3* (encoding A20), and *Hopx* compared with day 7 graft-infiltrating (CD45.1^+^) extravasated regulatory T cells (Supplemental Figure 6, B and C).

Similar to our observations for regulatory T cells, the transcriptional profile of day 21 graft-infiltrating (CD45.1^+^) extravasated CD8^+^ and non-regulatory CD4^+^ T cells resembled the respective day 7 graft-resident (CD45.2^+^) to a greater degree than day 7 graft-infiltrating (CD45.1^+^) extravasated T cell populations (Supplemental Figure 7). Compared with day 7 graft-infiltrating (CD45.1^+^) CD8^+^ T cells, day 7 graft-resident (CD45.2^+^) and day 21 graft-infiltrating (CD45.1^+^) CD8^+^ T cells had higher expression levels of *Pdcd1* (encoding PD-1), *Ctla4*, *Lag3* (encoding lymphocyte-activation gene 3), and *Il2rb* (encoding IL-2 receptor subunit β), markers associated with exhaustion and/or immunoregulation (Supplemental Figure 7B) (18, 19). We also examined the single-cell TCR profiles of graft-resident (CD45.2), graft-infiltrating (CD45.1), and splenic (CD45.1) Foxp3^+^ cells 4 days after retransplantation of tolerant BALB/c (CD45.2) lungs (previously transplanted into CSB-treated B6 [CD45.2] mice) into non-immunosuppressed B6 (CD45.1) recipients. Compared with graft-resident (CD45.2) Foxp3^+^ cells, we observed a greater clonal diversity in graft-infiltrating (CD45.1) and splenic (CD45.1) Foxp3^+^ cells (Supplemental Figure 8). Finally, when compared with graft-infiltrating Foxp3^+^ cells, graft-resident Foxp3^+^ cells were shifted from a central (CD44^hi^CD62L^hi^) to an effector memory (CD44^hi^CD62L^lo^) phenotype 7 days after retransplantation (Figure 3, E–I).

### Depletion of recipient Foxp3^+^ cells results in loss of allograft tolerance with development of AMR and ACR.

**Figure 4:**
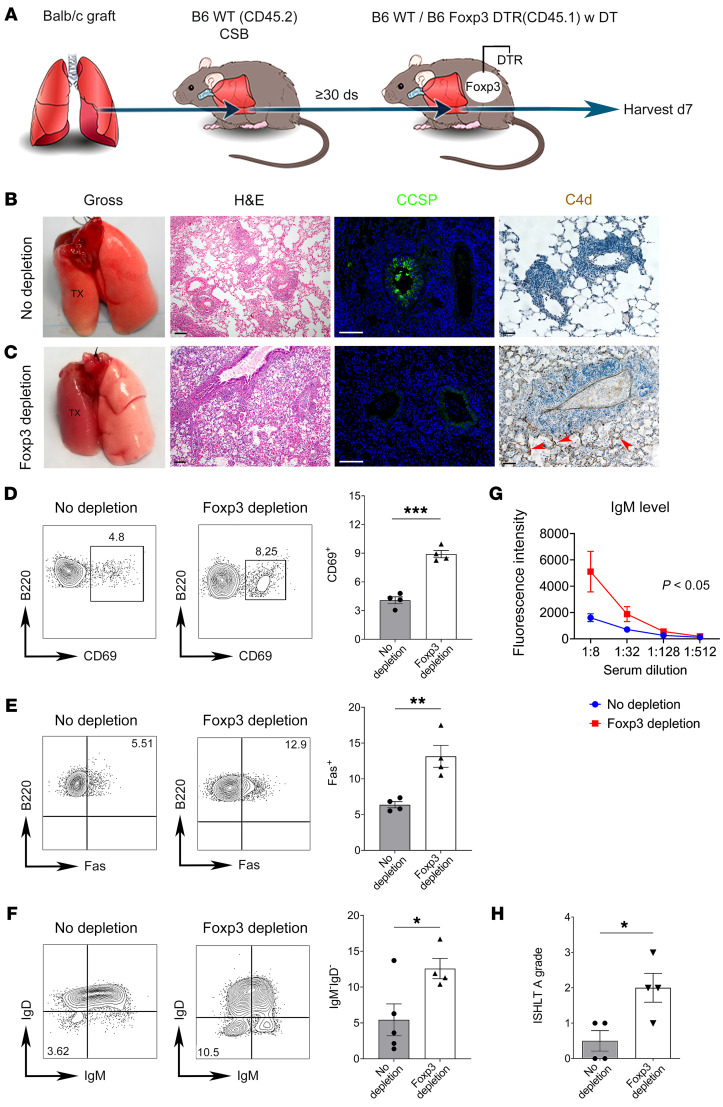
Depletion of recipient Foxp3^+^ cells results in loss of allograft tolerance. (**A**) Schematic depicting left lung from BALB/c (CD45.2) donor initially transplanted into CSB-treated B6 (CD45.2) primary recipient and then at least 30 days later retransplanted into non-immunosuppressed B6 (CD45.1) or B6 Foxp3-DTR (CD45.1) secondary recipient, with diphtheria toxin (DT) administration and analysis 7 days after retransplantation. (**B** and **C**) Gross image (first panel, *n* = 4), H&E histology (second panel, *n* = 4), CCSP immunofluorescence staining (green, third panel, *n* = 2), and immunohistochemical staining for complement 4d (C4d) (brown, fourth panel; arrows point to alveolar endothelial C4d staining; *n* = 2) in DT-treated B6 (**B**) and B6 Foxp3-DTR (**C**) secondary recipients. (**D**–**F**) Representative flow cytometric plots and quantification of abundance of CD69 (**D**), Fas (**E**), and IgM/IgD (**F**) expression in graft-infiltrating CD45.2^–^CD45.1^+^CD19^+^B220^+^ B cells in control (left panels) and recipient Foxp3-depleted (right panels) retransplants (*n* ≥ 4). (**G** and **H**) Flow cytometric analysis of serum donor-specific IgM antibody titers (expressed as mean fluorescence intensity) (**G**) and International Society for Heart and Lung Transplantation (ISHLT) A rejection grades (**H**) in control and recipient Foxp3-depleted retransplants (*n* = 4). Results expressed as mean ± SEM. Scale bars: 100 μm. *P < 0.05, **P < 0.01, ***P < 0.001. DT, diphtheria toxin; DTR, diphtheria toxin receptor; TX, transplanted lung.

To understand the role of recipient-derived graft-infiltrating Foxp3^+^ cells, we used B6 Foxp3 diphtheria toxin receptor (DTR) mice as secondary recipients of previously tolerized BALB/c lung allografts (Figure 4A). Treatment of these mice with diphtheria toxin (DT) after retransplantation allowed us to selectively deplete recipient Foxp3^+^ cells while preserving graft-resident Foxp3^+^ cells (Supplemental Figure 9). Control transplants where secondary B6 wild-type recipients were treated with DT were ventilated 7 days after retransplantation (Figure 4B). Consistent with previous reports, these allografts exhibited intact airspaces with preservation of BALT on histological examination (Figure 4B). Additionally, immunostaining revealed CCSP expression in the airways (Figure 4B). Finally, we examined complement 4d (C4d) deposition, a marker of complement activation, and found no positive staining in these control retransplanted lungs (Figure 4B). Altogether, these findings confirm our previous observations that lung allograft tolerance is maintained following retransplantation into a non-immunosuppressed B6 mouse, when Foxp3^+^ cells are present in the recipient (7). Conversely, when we depleted recipient Foxp3^+^ cells, the retransplanted lung was not ventilated (Figure 4C). We observed histological hallmarks of AMR, including neutrophilic capillaritis, flattening of the airway epithelium, obliterated alveolar spaces filled with hyaline membrane deposition, loss of CCSP expression, and endothelial C4d deposition (Figure 4C) (20). Compared with control retransplants, a higher proportion of graft-infiltrating recipient B cells in recipient Foxp3-depleted retransplants expressed CD69 and Fas (Figure 4, D and E) and underwent class-switch recombination as evidenced by lack of surface IgM and IgD expression (Figure 4F). Recipient Foxp3 depletion also resulted in higher serum levels of IgM donor-specific antibodies (DSAs) compared with control conditions (Figure 4G and Supplemental Figure 10). In addition, Foxp3 depletion resulted in concomitant ACR as evidenced by perivascular mononuclear infiltrates with endothelialitis (Figure 4, C and H). Thirty days after retransplantation of tolerant lung allografts, recipient Foxp3 depletion resulted in graft necrosis (Supplemental Figure 11). Collectively, these findings demonstrate that lung allografts lose their tolerant state and undergo changes consistent with AMR with concomitant ACR in the absence of continuous replenishment of Foxp3^+^ cells from the recipient.

### Rejection after recipient Foxp3^+^ cell depletion is dependent on B cells.

**Figure 5:**
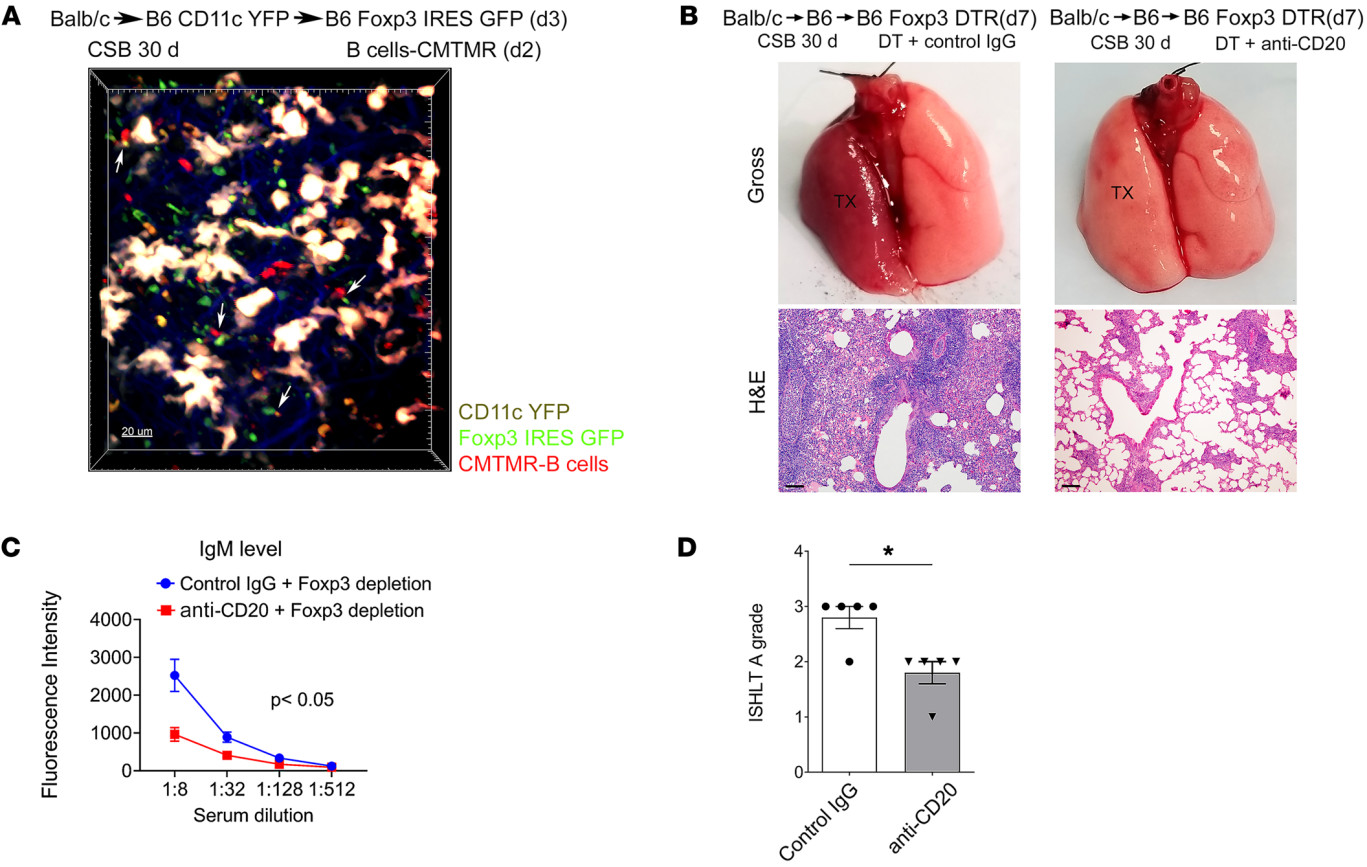
Rejection after recipient Foxp3^+^ cell depletion is dependent on B cells. (**A**) BALB/c lungs were initially transplanted into CSB-treated B6 CD11c-EYFP mice and then retransplanted into non-immunosuppressed B6 Foxp3-GFP hosts at least 30 days later. Recipient-matched B6 B cells labeled with the rhodamine-based red cell dye CMTMR were injected into recipients 2 days after retransplantation, and allografts were imaged with intravital 2-photon microscopy the following day (*n* = 3). White arrows point to contacts between Foxp3^+^ (green) and B cells (red). CD11c^+^ cells (yellow) mark the BALT within tolerant lung allografts (*n* = 4). Scale bar: 20 μm. (**B**) Gross (top) and histological (H&E; bottom) images of BALB/c lungs that were initially transplanted into CSB-treated B6 mice and then at least 30 days later retransplanted into Foxp3-depleted non-immunosuppressed B6 recipients that received anti-CD20 (right) or isotype control antibodies (left). Scale bars: 100 μm. (**C** and **D**) Flow cytometric analysis of serum IgM DSA titers (expressed as mean fluorescence intensity) (**C**) and ISHLT A rejection grades (**D**) in retransplant recipients described in **B** (*n* ≥ 4). DT, diphtheria toxin; DTR, diphtheria toxin receptor; TX, transplanted lung.

To further assess the role of B cells in rejection mediated by depletion of recipient Foxp3^–^ cells, we transplanted BALB/c lungs into CSB-treated B6 CD11c-EYFP mice. At least 30 days later, these tolerized allografts were retransplanted into non-immunosuppressed B6 Foxp3-GFP recipients. We adoptively transferred recipient-matched B6 9-[2-carboxy-4(or 5)-[[4-(chloromethyl)benzoyl]amino]phenyl]-3,6-bis(dimethylamino)-xanthylium–labeled (CMTMR-labeled) (red) B cells 2 days after retransplantation and imaged the lung grafts intravitally 1 day later. In areas of BALT characterized by CD11c^+^ cell aggregates, we observed prolonged interactions between recipient-derived Foxp3^+^ and B cells (Figure 5A and Supplemental Video 3). We next treated secondary non-immunosuppressed B6 recipients of tolerized BALB/c lung allografts with anti-CD20 antibodies to deplete B cells concurrently with the depletion of recipient Foxp3^+^ cells (Supplemental Figure 12). In contrast to recipients that received isotype control antibodies, B cell depletion substantially reduced inflammatory changes characteristic of AMR and significantly reduced serum levels of IgM DSAs 7 days after retransplantation (Figure 5, B and C). In addition, B cell depletion reduced the severity of ACR (Figure 5D). To gain additional mechanistic insight into the role of antibody production by recipient B cells in the rejection process in our model, we retransplanted tolerant lung allografts into B6 wild-type or activation-induced cytidine deaminase/secretory μ chain (AID/μS)–knockout mice treated with anti-CD25 antibody (PC61) (AID/μS–knockout mice have B cells that cannot secrete antibodies but can function as antigen-presenting cells) (Supplemental Figure 13). B6 AID/μS–knockout recipients did not form donor-specific IgM antibodies. Compared with PC61-treated B6 wild-type mice, allograft inflammatory changes and the severity of ACR were reduced after transplantation into AID/μS–knockout recipients. Thus, lung graft rejection induced by recipient Foxp3^+^ cell depletion is dependent on B cell–mediated immune responses.

### Lung allograft tolerance is maintained in the global absence of CD4^+^ T cells.

**Figure 6:**
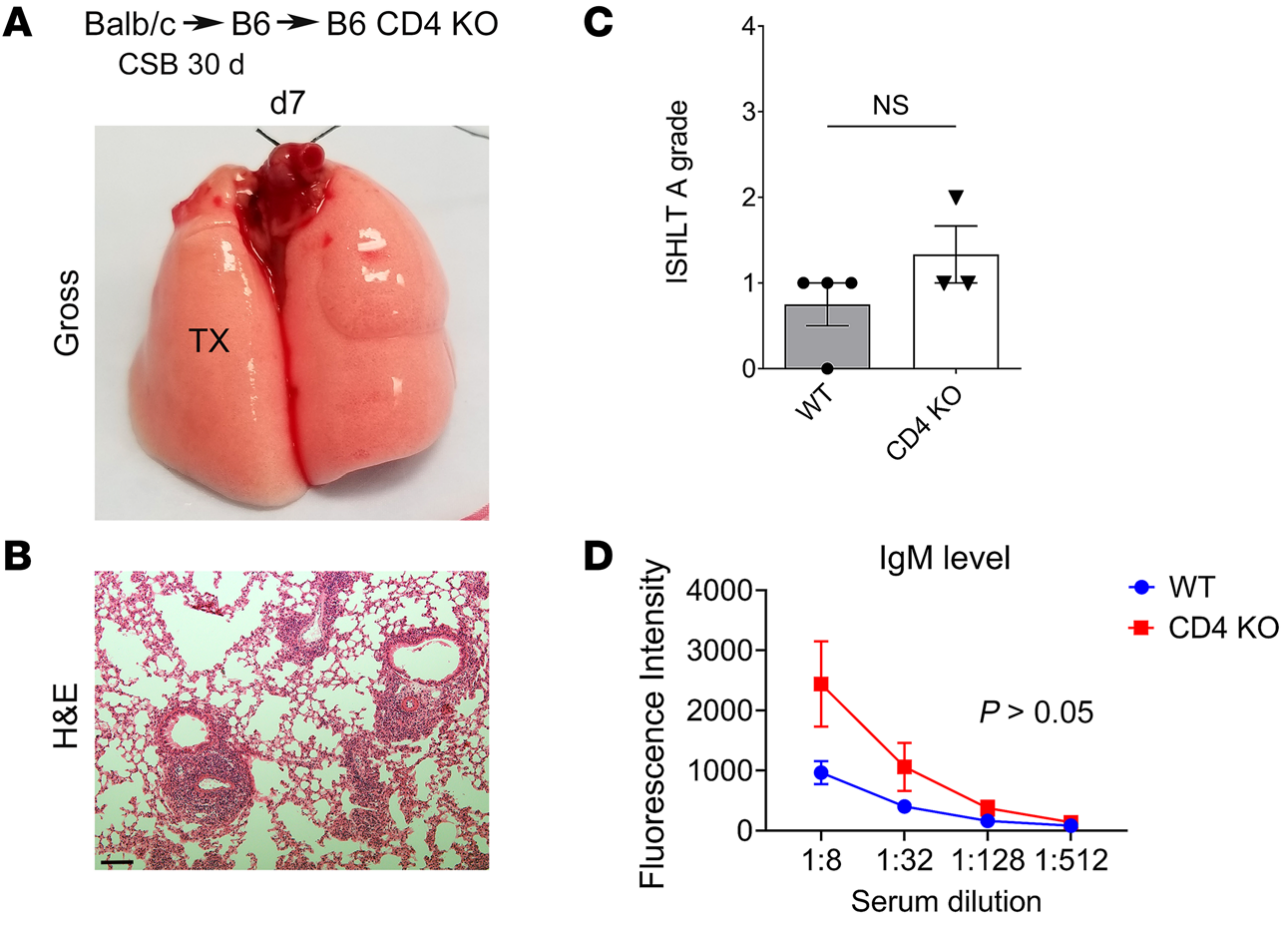
Lung allograft tolerance is maintained in the global absence of CD4^+^ T cells. (**A** and **B**) Gross (**A**) and histological (H&E) (**B**) images of BALB/c lungs that were initially transplanted into CSB-treated B6 mice and then at least 30 days later retransplanted into non-immunosuppressed B6 CD4 knockout recipients. Scale bar: 100 μm. (**C** and **D**) ISHLT A rejection grades (**C**) and flow cytometric analysis of serum IgM DSA titers (expressed as mean fluorescence intensity) (**D**) in retransplants depicted in **A** and **B** compared with tolerant BALB/c lung allografts that were retransplanted into non-immunosuppressed B6 mice (*n* ≥ 3). Mice were examined 7 days after retransplantation. TX, transplanted lung.

Our previous work has shown that recipient B cells require T cell help to mediate rejection when graft-resident Foxp3^+^ cells are depleted (6). Therefore, we next set out to examine the effect of complete absence of recipient CD4^+^ cells on the maintenance of tolerance. For this purpose, we transplanted BALB/c lungs into CSB-treated B6 recipients and then, at least 30 days later, retransplanted these tolerized grafts into B6 CD4-deficient secondary hosts. CD4-deficient mice lack conventional CD4^+^ T cells in addition to regulatory CD4^+^Foxp3^+^ cells. At both 7 days (Figure 6, A–C) and 30 days (Supplemental Figure 14) after retransplantation, lung allografts were ventilated, and the graft architecture remained intact with preservation of BALT. In the absence of recipient CD4^+^ cells, serum levels of IgM DSAs did not differ significantly from those seen with control retransplants in wild-type secondary recipients (Figure 6D).

### IL-33 promotes the expansion and activation of lung allograft–resident Foxp3^+^ cells.

**Figure 7:**
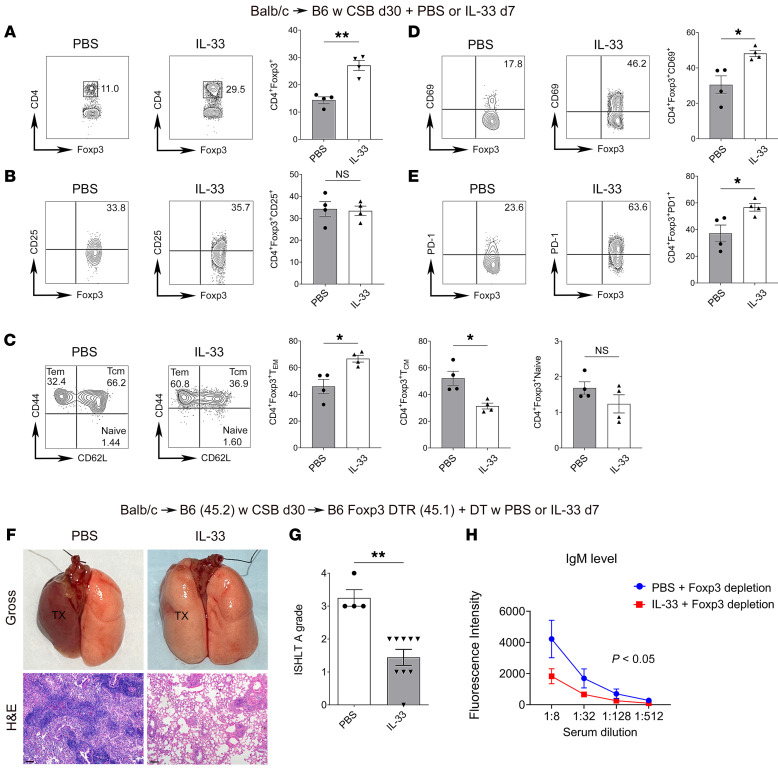
IL-33 promotes the expansion and activation of lung allograft–resident Foxp3^+^ cells. BALB/c lungs were transplanted into CSB-treated B6 recipients. At least 30 days after transplantation, recipients were treated with intratracheal IL-33 or PBS, and grafts were analyzed 7 days later. (**A**–**E**) Representative flow cytometry plots and quantification of abundance of Foxp3-expressing CD45^+^CD90.2^+^CD4^+^CD8^–^ T cells (**A**), CD25-expressing CD45^+^CD90.2^+^CD4^+^CD8^–^Foxp3^+^ T cells (**B**), effector memory (CD44^hi^CD62L^lo^), central memory (CD44^hi^CD62L^hi^), and naive (CD44^lo^CD62L^hi^) CD45^+^CD90.2^+^CD4^+^CD8^–^Foxp3^+^ T cells (**C**), CD69-expressing CD45^+^CD90.2^+^CD4^+^CD8^–^Foxp3^+^ T cells (**D**), and (**E**) PD-1–expressing CD45^+^CD90.2^+^CD4^+^CD8^–^Foxp3^+^ T cells in lung allografts after treatment with IL-33 or PBS (*n* = 4). (**F**) Gross (top) and histological (H&E; bottom) appearances of left BALB/c lungs that were initially transplanted into CSB-treated B6 recipients and then 30 days later retransplanted into DT-treated B6 Foxp3-DTR secondary recipients that received PBS (left; *n* = 4) or IL-33 (right; *n* = 9) intratracheally. Grafts were examined 7 days after retransplantation. Scale bars: 100 μm. (**G** and **H**) ISHLT A rejection grades (**G**) and flow cytometric analysis of serum IgM DSA titers (expressed as mean fluorescence intensity) (**H**) in recipients depicted in **F** (PBS, *n* = 4; IL-33, *n* = 9). Results expressed as mean ± SEM. *P < 0.05, **P < 0.01. DT, diphtheria toxin; DTR, diphtheria toxin receptor; Tcm, T central memory; Tem, T effector memory; TX, transplanted lung.

IL-33 is a cytokine that drives Th2 immunity and activation of innate lymphoid cells (21, 22), but it has also been shown to be critical in maintaining homeostasis of tissue-resident regulatory T cells (23, 24). To study the effect of exogenous IL-33 administration in our model, we transplanted BALB/c lungs into CSB-treated B6 recipients. At least 30 days later, we administered recombinant IL-33 or phosphate-buffered saline (PBS) control intratracheally and examined the T cell composition in the lung allografts 7 days later. We observed a significant expansion of the CD4^+^Foxp3^+^ T cell population in IL-33–treated mice when compared with PBS controls (Figure 7A). While IL-33 injection did not alter the proportion of CD4^+^Foxp3^+^ cells that expressed CD25, it did increase the proportion of effector memory CD4^+^Foxp3^+^ cells (Tem, CD44^hi^CD62L^lo^), decreased central memory CD4^+^Foxp3^+^ cells (Tcm, CD44^hi^CD62L^hi^), and resulted in a higher proportion of CD4^+^Foxp3^+^ cells expressing CD69 and PD-1 (Figure 7, B–E). IL-33 treatment did not have a significant effect on the memory phenotype of graft-resident CD8^+^ T cells (Supplemental Figure 15). We next wanted to determine whether intratracheal IL-33 administration expands CD4^+^Foxp3^+^ cells in the allograft by directly acting on Foxp3^+^ cells. To this end, we transplanted BALB/c lungs into CSB-treated B6 Foxp3-YFP-Cre *St2^fl/fl^* or B6 Foxp3-YFP-Cre controls. There was significantly less expansion of intragraft Foxp3^+^ cells following intratracheal treatment with IL-33 when Foxp3^+^ cells did not express ST2 (Supplemental Figure 16). Thus, having observed that local administration of IL-33 induces the expansion and activation of graft-resident CD4^+^Foxp3^+^ cells, we next set out to examine whether this treatment prevents rejection when graft-resident Foxp3^+^ cells are not replenished. To this end, we transplanted BALB/c lungs into CSB-treated B6 recipients. At least 30 days later these tolerized allografts were retransplanted into non-immunosuppressed DT-treated B6 Foxp3-DTR mice. We administered recombinant IL-33 or PBS into their airways and examined the mice 7 days later. We found that intratracheal IL-33 therapy reduced the inflammatory changes characteristic of AMR and severity of ACR (Figure 7, F and G). In comparison with control PBS-treated retransplants, IL-33 administration resulted in significant reductions of serum IgM DSA levels (Figure 7H).

## Discussion

Mechanisms of tolerance after solid organ transplantation differ by organ. Our observations extend previous findings from our laboratory and others that immunoregulatory pathways are established locally in the lung graft (6, 7, 17, 25). In this study, we show that the preservation of graft-resident Foxp3^+^ cells is critical for the maintenance of lung allograft tolerance. Using a mouse lung retransplant model, we demonstrate that the pool of tissue-resident Foxp3^+^ cells is maintained through continuous replenishment with Foxp3^+^ cells that are recruited from the periphery to the graft. Waldmann’s group described the concept of self-sustaining “infectious tolerance” in skin transplant models, where Foxp3^+^ cells can induce the generation of new Foxp3^+^ cells from naive T cells (26, 27). By contrast, our study shows that Foxp3^+^ cells that are recruited to tolerant lung allografts are not derived from peripheral conversion of non-regulatory T cells. We demonstrate that graft-resident Foxp3^+^ cells are largely of thymic origin and have distinct features that have been described for regulatory T cells residing in various non-lymphoid tissues (28–30). Finally, our experiments suggest that lung graft–resident Foxp3^+^ cells can potentially be targeted with inhaled immunomodulatory agents.

Our group has previously reported that tolerance induction after lung transplantation depends on production of IFN-γ by central memory CD8^+^ T cells, which drives the production of nitric oxide (3). We showed that at 7 days after transplantation, the proportion of intragraft CD4^+^ T cells expressing Foxp3 was lower when recipients lacked CD8^+^ T cells. While future studies will need to explore this association mechanistically, reports exist that IFN-γ and nitric oxide can promote the expansion of regulatory CD4^+^Foxp3^+^ T cells (31, 32). Here, we show that graft-resident CD8^+^ T cells in long-term tolerant lung allografts express markers that are characteristic of exhaustion and/or immunoregulation. This observation is consistent with a recent report showing that CD8^+^ T lymphocytes that infiltrate spontaneously accepting mouse kidney allografts are reprogrammed to exhausted/regulatory-like cells (19).

AMR has been increasingly recognized as a cause of severe graft dysfunction after lung transplantation and also as a risk factor for the development of chronic lung allograft dysfunction (2). To this end, we and others have previously reported that humoral alloimmune responses are critical drivers of chronic fibrotic remodeling after lung transplantation (33, 34). We have shown that depletion of graft-resident Foxp3^+^ cells results in the local activation of B cells and AMR (6). The use of a mouse lung retransplant model enabled us to selectively deplete graft-infiltrating Foxp3^+^ cells while preserving graft-resident Foxp3^+^ cells (35). We demonstrate that in the absence of continuous recruitment of Foxp3^+^ cells to the transplanted lung, graft-resident Foxp3^+^ cells alone are not sufficient to prevent rejection. Histologically, rejected allografts have features characteristic of AMR, including a pattern of acute lung injury with hyaline membrane formation, neutrophilic capillaritis, loss of CCSP expression, and complement deposition along with ACR (20). A case series from our center reported ACR in 29% of human lung transplant recipients who were diagnosed with C4d-positive AMR (36). However, given potential sampling errors associated with transbronchial biopsies, the incidence of concomitant AMR and ACR in humans may be higher. We show that graft-infiltrating Foxp3^+^ cells interact with newly recruited B cells, and B cell depletion reduces the severity of AMR and ACR. These observations raise the possibility that — in addition to mediating AMR through production of alloantibodies — B cells may exacerbate ACR through their function as antigen-presenting cells. While we and others have previously used anti-CD25 antibodies to deplete regulatory T cells, we recognize that this approach has some limitations, including an incomplete depletion due to the presence of Foxp3^+^CD25^lo^ cells and an inability to target donor versus recipient regulatory T cells (7, 37, 38). Nevertheless, the reduction in severity of ACR after treatment of AID/μS–deficient recipients with PC61 raises the possibility that antibody production by B cells, directly or indirectly, may contribute to the activation of T cells (39, 40).

Unlike what occurs with selective depletion of recipient Foxp3^+^ cells, global absence of recipient CD4^+^ cells did not trigger rejection. We have previously reported that the generation of DSAs is inhibited and graft rejection is attenuated after depletion of graft-resident Foxp3^+^ cells when recipients lack T cells or when CXCL13, the ligand for CXCR5, is neutralized (6). These findings support the notion that B cell activation in the context of graft rejection mediated by Foxp3^+^ cell depletion requires CD4^+^ T cell help, which is presumably provided by graft-infiltrating CD4^+^CXCR5^+^ T follicular helper cells.

Phenotypic and transcriptional differences between graft-resident and newly recruited Foxp3^+^ cells suggest that their functions are imprinted within the transplanted lung. This notion is further supported by their clonal expansion in the graft, a finding that mirrors observations for tissue-resident regulatory T cells in other locations (29, 30). Previous work has proposed a two-step model for the acquisition of the phenotype of regulatory T cells that reside in visceral adipose tissue, where an initial priming step in the spleen is followed by further education in the non-lymphoid tissue (41). In previous work we have shown that Foxp3^+^ cells leave the BALT within tolerant lung allografts via lymphatics and suppress alloimmune responses in the periphery to donor-matched but not third-party grafts (17). Previous studies have shown that transplantation of vascularized donor thymic tissue along with an allograft results in downregulation of alloimmune responses (42). While several mechanisms have been proposed, the beneficial effects of thymic grafting on allograft survival could — at least in part — be due to migration of regulatory T cells from the thymic graft to the periphery (43). Our observations suggest that the suppressive capacity of Foxp3^+^ cells, both locally and systemically, is dependent on alloantigen recognition and clonal expansion in the allograft, which likely occurs within the BALT. The requirement for continuous replenishment of the allograft tissue–resident Foxp3^+^ cell population raises several not mutually exclusive possibilities. Tissue-resident Foxp3^+^ cells could have a limited lifespan, which could be a function of insufficient levels of cytokines within the BALT that promote their survival. Foxp3^+^ cells could become unstable within the graft environment and lose their suppressive function. Finally, Foxp3^+^ cells could continuously egress from the graft and migrate to the periphery; however, this is not likely to be a dominant mechanism in our experimental system, as lymphatic drainage from the allograft is not reestablished until several weeks after transplantation (17, 44).

In addition to their clonal expansion, important similarities exist between Foxp3^+^ cells that reside within tolerant lung allografts and regulatory T cells that have been described and characterized in other non-lymphoid tissues. For example, regulatory T cells within visceral adipose tissue express high levels of Helios and neuropilin-1, and Foxp3^–^ cells do not convert into Foxp3^+^ cells, indicating that these cells are likely of thymic origin (28). Similarly, regulatory T cells that accumulate in injured skeletal muscles express Helios and neuropilin-1 (30). Moreover, the transcriptome of muscle-resident regulatory T cells is distinct from that in regulatory T cells that reside in secondary lymphoid organs. Notably, similar to our observations in tolerant lung allografts, regulatory T cells that reside in skeletal muscle and visceral adipose tissue express ST2, the receptor for IL-33 (28, 30).

IL-33 is a proinflammatory IL-1 family cytokine expressed at epithelial tissue barriers and a nuclear alarmin that alerts innate immune cells and Th2 lymphocytes to injury or infection (45). This cytokine is also known to promote type 1 immune responses against tumors (46). However, IL-33 plays important roles in downregulating deleterious immune responses (47). For example, our group has recently reported that ischemia/reperfusion injury after lung transplantation increases the expression of IL-33 in airway epithelial cells (48). IL-33 drives IL-5 production by innate lymphoid cells type 2, which facilitates the recruitment of eosinophils and tolerance induction. We show that local administration of IL-33 expands and activates lung allograft–resident Foxp3^+^ cells. This observation extends findings from several studies that have demonstrated the capacity of IL-33 to expand tissue-resident regulatory T cells in various sites (23, 24, 28, 49–51). IL-33 can mediate its effect by signaling through several pulmonary cell populations. For example, a recent study showed that IL-33–ST2 signaling in macrophages promotes club cell repair after naphthalene-induced injury (52). We show that IL-33–mediated expansion of Foxp3^+^ cells in lung allografts is — at least in part — dependent on *St2* expression by Foxp3^+^ cells. We also demonstrate that intra-airway IL-33 administration reduces the severity of AMR and ACR at 7 days after retransplantation when recruitment of Foxp3^+^ cells to the allograft is prevented. Our observations extend previous findings that IL-33 treatment prolongs the survival of murine cardiac and skin allografts, an effect that is dependent on regulatory T cells (53, 54).

IL-33 has also been reported to potentially contribute to various lung diseases, including pulmonary fibrosis, asthma, and chronic obstructive pulmonary disease (55–57). While we show beneficial effects at 7 days after retransplantation, additional long-term studies are required to assess the efficacy and safety of local IL-33 administration in lung transplant recipients. Nevertheless, the critical role that tissue-resident Foxp3^+^ cells play in maintaining tolerance after lung transplantation provides a compelling rationale for exploring local therapies, especially since their proximity to airways due to their accumulation in the BALT renders them accessible to inhaled agents. Local immunosuppressive therapies are appealing for lung transplant patients as they may decrease the harmful side effects of systemic therapy (58). We have recently reported that cyclosporine-based immunosuppression triggers rejection of tolerant lung allografts (59). Our flow cytometry and gene expression data indicate that a portion of tissue-resident Foxp3^+^ cells in tolerant lung allografts expresses CD25, the α subunit of the IL-2 receptor. As cyclosporine is known to suppress IL-2, studies are warranted that examine the effect of local IL-2 administration on lung transplant tolerance. A recent study showed that systemic treatment of mouse lung transplant recipients with IL-2/anti–IL-2 antibody complexes prior to engraftment facilitated long-term survival, which was dependent on accumulation of Foxp3^+^ cells in the allograft (25).

The continuous replenishment of Foxp3^+^ cells from peripheral sites points to therapeutic strategies in addition to the local administration of cytokines that promote their survival and activation. To this end, an important area for future investigation is to define chemotactic gradients that guide recipient Foxp3^+^ to the BALT within tolerant lung allografts. Future studies could focus on the role of CXCL12, as we observed that, compared with graft-resident Foxp3^+^ cells, recently recruited Foxp3^+^ cells express higher levels of CXCR4. Interestingly, tolerance after vascularized composite allotransplantation is dependent on the presence of CXCR4-expressing Foxp3^+^ cells in the bone marrow, which may at least in part be due to guiding of the accumulation of regulatory T cells in specific niches (60). Requirements for specific chemotaxins may differ between transplanted tissues. For example, tolerance after rat kidney transplantation is associated with production of CCL5 by myeloid-derived suppressor cells, which mediates the recruitment of regulatory T cells to the graft (61). CCL22 overexpression in pancreatic islet transplants attracts regulatory T cells and protects the grafts (62).

One noteworthy gene that is highly expressed by tissue-resident Foxp3^+^ cells in tolerant lung allografts is amphiregulin, a growth factor that signals through EGFR. Amphiregulin plays an important role in tissue homeostasis and repair after injury. In a model of pulmonary hypertension, amphiregulin produced by endothelial cells is thought to signal in an autocrine fashion to promote endothelial cell survival and proliferation (63). Notably, in a mouse model of respiratory viral infection, regulatory T cells play a critical role in tissue repair via their production of amphiregulin that is distinct from their immunosuppressive role (64). In skeletal muscle, amphiregulin signals to muscle satellite cells and myoblasts for muscle regeneration after injury. Ablation of Foxp3^+^ cells compromises muscle repair, whereas exogenous amphiregulin administration to Foxp3^+^ cell-ablated mice was associated with upregulation of muscle repair genes and enhanced myogenic differentiation of satellite cells (30). While some studies have shown that amphiregulin enhances the suppressive capacity of regulatory T cells, EGFR expression was largely restricted to stromal cells in our lung grafts (65, 66). Thus, the severe inflammation that we observe when recipient Foxp3^+^ cells lack *Areg* suggests that Foxp3-derived amphiregulin maintains a homeostatic state by directly acting on intragraft stromal cell populations.

In conclusion, we characterize a graft-resident Foxp3^+^ cell population following lung transplantation that is critical in maintaining tolerance. These cells accumulate within BALT where they acquire unique features and need to be continually replenished by recipient-derived Foxp3^+^ cells. Our findings highlight the importance of preserving this cell population in lung allografts, which may be achieved through local therapy.

## Methods

### Sex as a biological variable.

Our study examined male and female animals, and similar findings are reported for both sexes. For transplants involving DT-mediated Foxp3 depletion, we used sex-matched combinations with hemizygous Foxp3-DTR males.

### Mice and reagents.

Mouse strains including C57BL/6J (B6 CD45.2), B6.SJL-PtprcaPepcb/BoyJ (B6 CD45.1), BALB/cJ, B6.Cg-Tg(Itgax-Venus)1Mnz/J (B6 CD11c-EYFP), B6 Foxp3tm9(EGFP/cre/ERT2)Ayr/J (B6 Foxp3–internal ribosome entry sites [IRES]–GFP) (referred to as B6 Foxp3-GFP), B6-Foxp3tm1Flv/J (B6 Foxp3-IRES-RFP) (referred to as B6 Foxp3-RFP), B6-Tg(Foxp3-HBEGF/EGFP)23.2Spar/Mmjax (B6 Foxp3-DTR), and B6.129S2-Cd4tm1Mak/J (B6 CD4KO) were purchased from The Jackson Laboratory. B6 CD11c-EYFP/Foxp3-IRES-GFP mice were generated by crossing of B6 CD11c-EYFP mice with B6 Foxp3-IRES-GFP mice. B6 Foxp3-YFP-Cre *Areg^fl/fl^*, B6 Foxp3-YFP-Cre *St2^fl/fl^*, and B6 Foxp3-YFP-Cre littermate controls were provided by HR Turnquist (University of Pittsburgh). B6 AID/μS–deficient (activation-induced cytidine deaminase/secretory μ chain–deficient) mice were originally provided by G. Chalassani (University of Pittsburgh) after permission from F. Lund (University of Alabama, Birmingham, Alabama, USA) and maintained in our colony. Six- to ten-week-old sex-matched mice were used for transplant procedures. After primary lung transplant, CSB was administered to the recipient mouse with anti-CD40 ligand (250 μg i.p.) and CTLA4-Ig (200 μg i.p.) on postoperative days 0 and 2, respectively (Bio X Cell). DT (Sigma-Aldrich) was administered into retransplant recipients (250 ng i.p. on postoperative days 0, 1, and 4 and then weekly as indicated). For select experiments, 250 μg of anti-CD20 neutralizing antibody (Bio X Cell) was administered i.v. to the recipient 1 day before retransplantation, and then i.p. on postoperative days 0, 1, and 4 after retransplantation, with IgG2c isotype serving as control (Bio X Cell). For some experiments, PC61 (Bio X Cell) was administered i.p. to retransplant recipients on days 0 (500 μg), 1, and 3 (250 μg each). Three doses of recombinant mouse IL-33 (50 μg/50 μL) (BioLegend) were administered into the airways of primary recipients (≥30 days after transplantation as well as 1 and 4 days later) and retransplant recipients (days 0, 1, and 4), with PBS serving as control.

### Surgical procedure.

Orthotopic left lung transplants and retransplants were performed, as previously described (35, 67).

### Flow cytometry.

Lung digestion and preparation of single-cell suspension were performed as previously described (3). Flow cytometry antibodies used were as follows: CD4 (clone GK1.5), CD44 (IM7), and CD69 (H1.2F3) (BioLegend); Foxp3 (FJK-16s), CD62L (MEL-14), Helios (22F6), PD-1 (J43), IgM (II/41), CD45.1 (A20), and CD19 (1D3) (Invitrogen); CD25 (PC61), CD45R (RA3-6B2), CD95 (Jo2), IgD (11-26c.2a), CD8a (53-6.7), CD45.2 (104), CD90.2 (53-2.1), and CD45 (30-F11) (BD Biosciences); and CD304 (neuropilin-1) (3DS304M) (eBioscience). Serum IgM DSA titers were determined with polyclonal fluorochrome-conjugated goat anti-mouse IgM (μ chain specific), as previously described (Jackson ImmunoResearch) (6). Differences in serum DSA levels between groups were determined at serum dilutions of 1:8. Cells were analyzed on a FACScan (BD) and Attune NxT Acoustic Focusing Cytometer (Thermo Fisher Scientific). Acquired flow cytometric data were analyzed with FlowJo v10.9 (FlowJo, BD). Live gates defined by forward and side scatter characteristics were used for analyses.

### Cell isolation and adoptive transfer.

For B cell adoptive transfer experiments, B cells were isolated from spleens of naive B6 mice using a B cell isolation kit (Miltenyi Biotec). Cells were stained with CMTMR (Thermo Fisher Scientific), and 2 × 10^6^ B cells were injected i.v. into recipient mice. For Foxp3-GFP^–^ cell adoptive transfer experiments, live CD45^+^CD90^+^CD4^+^GFP^–^ cells were purified by fluorescence-activated cell sorting from spleen and lymph nodes of B6 Foxp3-IRES-GFP (CD45.1) mice using a MoFlo sorter (BD), and 5 × 10^6^ cells were injected into the recipient mouse via the right internal jugular vein.

### Intravital 2-photon microscopy.

Lung grafts were imaged by intravital 2-photon microscopy, as previously described (11). Twenty microliters of 655-nm nontargeted quantum dots (q-dots, Life Technologies) in 50 μL of PBS or 8 μL of dextran–rhodamine B (30,000MV, Invitrogen) suspended in 150 μL of PBS were injected i.v. to label blood vessels. CMTMR-labeled B cells were isolated and injected 2 days after retransplantation into lung recipients and 24 hours before imaging. Grafts were stabilized within the left chest and imaged with a custom 2-photon microscope using SlideBook v6 (Intelligent Imaging Innovations Inc.). Sequential *Z*-sections (2.0 μm each) were acquired, yielding an imaging volume of 330 × 330 × 40 μm^3^. Videos and images were acquired and processed with Imaris 9.7.2 (Bitplane).

### Histology.

Transplanted and retransplanted lungs were fixed in formaldehyde, embedded in paraffin, and sectioned into 5 μm slices. Lung sections were stained with hematoxylin and eosin and assessed for antibody-mediated rejection and graded for acute cellular rejection (International Society for Heart and Lung Transplantation [ISHLT] A grades) by a blinded pathologist.

### Immunofluorescence and immunohistochemistry.

Formalin-fixed, paraffin-embedded 5 μm mouse lung sections were deparaffinized and rehydrated. Antigen retrieval was performed in a pH 6.0 citrate-based buffer (Vector Laboratories) at 100°C for 20 minutes, followed by three 5-minute washes in PBS with 1% Tween (PBS-T). For immunofluorescent staining, nonspecific binding was blocked with 10% normal goat serum (Invitrogen). Slides were costained with rabbit anti-mouse polyclonal CCSP (1:250; Seven Hills Bioreagent, catalog WRAB-3950) and incubated overnight at 4°C. Slides were washed with PBS-T and then incubated with goat anti-rabbit Alexa Fluor 488 (Thermo Fisher Scientific, catalog A-11008) for 45 minutes. After three PBS-T washes, the slides were counterstained with nuclear stain Hoechst 33342 (1:1,000; Invitrogen, catalog H3570) for 15 minutes. Slides were coverslipped with aqueous mounting medium (Invitrogen). For immunohistochemical staining, endogenous hydrogen peroxidase and alkaline phosphatase were quenched with BLOXALL endogenous blocking solution (Vector Laboratories), nonspecific protein binding was blocked with 10% goat serum (Vector Laboratories), and nonspecific avidin/biotin binding was blocked with Avidin/Biotin block (Vector Laboratories). Slides were stained with rabbit anti-mouse polyclonal C4d (1:100; Hycult Biotech, catalog HP8033) overnight at 4°C, washed, and then stained with biotinylated goat anti-rabbit IgG (1:200; Vector Laboratories, catalog BA-1000-1.5) for 30 minutes. Antibody binding was amplified with the VECTASTAIN ABC Kit (Vector Laboratories) and visualized with peroxidase substrate 3,3-diaminobenzidine (Vector Laboratories). Slides were counterstained with hematoxylin (Leica Biosystems) and coverslipped with non-aqueous mounting medium (Thermo Fisher Scientific). Human slides were stained with 1:200 rat anti–human Foxp3 (clone PCH101, eBioscience) at room temperature for 2 hours, then amplified and visualized with the VECTASTAIN ABC Kit, Peroxidase (Rat IgG) (Vector Laboratories). The slides were incubated with 1:200 polyclonal rabbit anti-amphiregulin (Bioss, catalog bs-3847R) overnight and then amplified and visualized with the VECTASTAIN ABC Kit, Alkaline Phosphatase (Rabbit IgG) (Vector Laboratories). All images were acquired using an Olympus BX61 microscope with a digital camera and CellSens Dimension software (version 1.18, Olympus).

### Single-cell suspension preparation for single-cell RNA sequencing.

For comparison of Foxp3^+^ cells in tolerant lung allografts and spleen, transplanted left lungs and recipient spleens were collected at least 30 days after transplantation of BALB/c lungs into CSB-treated B6 Foxp3-GFP recipients. Lung tissue was digested, and single-cell suspensions were prepared as previously described (68). Cells were stained using antibodies specific for CD45, CD90.2, CD4, CD8a, and 4′,6-diamidino-2-phenylindole (DAPI) (BD Biosciences) and were sorted by flow cytometry for DAPI^–^CD45^+^CD90.2^+^CD4^+^CD8a^–^GFP^+^ cells into a 250 μL cell resuspension buffer (0.04% bovine serum albumin in PBS). Collected cells were centrifuged (300 relative centrifugal force for 5 minutes at 4°C) and resuspended in collection buffer to a target concentration of 1,000 cells/μL. Cells were counted on a hemocytometer before proceeding. For comparison of graft-resident versus graft-infiltrating T cells, lungs from BALB/c donors transplanted into CSB-treated B6 (CD45.2) primary recipients, and then retransplanted into B6 (CD45.1) secondary recipients at least 30 days later, were harvested at 7 or 21 days after retransplantation. A fluorochrome-labeled anti-CD45.1 antibody was injected i.v. (CD45.1-i.v.) into recipient mice 5 minutes before sacrifice to allow for exclusion of intravascular cells. Cells were stained using antibodies specific for CD90.2, Lin (CD19, NK1.1, CD11b, Ly6G), CD45.2, CD45.1, and DAPI (BD Biosciences) and were sorted by flow cytometry for DAPI^–^CD90.2^+^Lin^–^CD45.2^+^CD45.1^–^ or DAPI^–^CD90.2^+^Lin^–^CD45.2^–^CD45.1^+^CD45.1-i.v.^–^ cells. For analysis of TCR clonotypes in regulatory T cells in retransplant recipients, lungs from BALB/c donors transplanted into CSB-treated B6 (CD45.2) primary recipients, and then retransplanted into B6 (CD45.1) secondary recipients at least 30 days later, were harvested along with recipient spleens 4 days after retransplantation. Cells were stained using antibodies specific for CD90.2, Lin (CD19 [clone 1D3, eBioscience], NK1.1 [clone PK136, BioLegend], CD11b [clone M1/70, Invitrogen], Ly6G [clone 1A8, BD Biosciences]), CD4, CD8a, CD45.2, CD45.1, and DAPI (BD Biosciences) and were sorted by flow cytometry for DAPI^–^Lin^–^CD45.2^+^CD45.1^–^CD90.2^+^CD4^+^CD8a^–^ or DAPI^–^Lin^–^CD45.2^–^CD45.1^+^CD90.2^+^CD4^+^CD8a^–^ cells. Single-cell suspensions were prepared as described above.

### Library preparation for single-cell RNA sequencing and TCR profiling.

Single-cell suspensions were submitted to the Genome Technology Access Center core facility (Washington University) for single-cell genome-scale metabolic model (GEM) construction and complementary DNA synthesis and library construction. For the sorted Foxp3^+^ cells, cells were labeled using TotalSeq anti-mouse hashtag antibodies (BioLegend) before sample submission. For the primary transplants, 2 sets of hashtagged samples were submitted for genome sequencing and TCR profiling. Samples were processed using the Chromium Single Cell 3′ Library & Gel Bead Kit (v3, 10x Genomics) and Chromium Single Cell V(D)J Reagent Kits (10x Genomics) following the manufacturer’s protocols. The libraries were sequenced on NovaSeq S4 (Illumina) targeting 50,000 reads per cell and 500 million read pairs per library. Cells were aligned to the mouse mm10-2020-A transcriptome using CellRanger (v6.1.1, 10x Genomics) to generate feature-barcoded count matrices. For TCR analysis of retransplants, we used the Chromium Next GEM Single Cell 5′ Kit (v2, 10x Genomics) with paired TCR sequencing.

### Analysis pipeline for single-cell RNA sequencing and TCR profiling.

Analysis was performed using the R Seurat v4.0.0 package. The following quality control steps were performed to filter the count matrices: (a) Cells expressing fewer than 500 genes were removed. (b) Cells expressing over 10,000 genes were discarded, as these could be potential multiplet events where more than a single cell was encapsulated within the same barcoded GEM. (c) Cells with more than 5% mitochondrial content were filtered out, as these were deemed to be of low quality (69). After quality control, normalization and variance stabilization of raw counts were performed using SCTransform, and cell cycle scores were computed and regressed out in combination with percentage mitochondrial reads (70). The normalized R object was then used for subsequent clustering and differential expression testing, and integration with Harmony was performed (71). Briefly, clusters were annotated into major cell populations, each major cell type was subsetted and renormalized, and principal component analysis and uniform manifold approximation and projection (UMAP) embedding, clustering, and differential expression analysis were performed. Clusters that resembled low-quality cells or doublets were removed from subsequent analysis. We used the FindAllMarkers function in Seurat to perform differential expression testing and annotated clusters into distinct cell types based on canonical gene markers. For downstream TCR analysis in primary transplants, TCRs with only one pair of productive rearrangements for TCRα and TCRβ chains were selected. Clonotypes in each sample were defined based on identical CDR3 nucleotide sequences together with identical V and J genes in both TCRα and TCRβ chains. Gini coefficients in each sample were calculated using the Gini function from the DescTools package v0.99.50. Shared clonotypes were defined as clonotypes coming from different tissues within the same mouse and containing identical CDR3 nucleotide sequences together with identical V and J genes. Visualization was performed with ggalluvial package v0.12.5. For analysis of TCR clonotypes in CD4^+^Foxp3^+^ regulatory T cells in retransplant recipients, the scRepertoire v2.2.1 package was used in R. Briefly, filter contig outputs from the 10x Genomics CellRanger pipeline were used to assign clonotype data based on TCRα and TCRβ chains. The clonalCompare function was used to visualize relative clonotypes across conditions. Clonal diversity was calculated using the Gini-Simpson index with 20 bootstraps.

### Sample preparation for single-nuclear RNA sequencing.

Lung grafts from 2 mice per experimental group were harvested, embedded in optimal cutting temperature (OCT) compound, and flash-frozen in liquid nitrogen. OCT-embedded frozen tissue samples were processed for nuclei extraction by the Tissue Procurement Core at Washington University. Briefly, tissue from each sample was processed using the gentleMACS Octo Dissociator and cell lysis performed using Nuclei Extraction Buffer Protocol (Miltenyi Biotec). Nuclei suspensions from 2 mice per group were pooled, counted, and resuspended at 1,500 nuclei/μL in 1× PBS with 1% bovine serum albumin and 0.2 U/μL RNase inhibitor. Pooled nuclei suspensions were encapsulated with barcoded oligo-dT–containing gel beads with the Chromium Single-Cell 3′ Library & Gel Bead Kit (v3, 10x Genomics) at the Genome Technology Access Center at Washington University. Libraries were sequenced on the NovaSeq S4 (Illumina), with a target of 50,000 reads per cell and 500 million read pairs per library.

### Statistics.

Data were analyzed using Prism (v10.0.3, GraphPad Software) and presented graphically as mean ± SEM. Groups were compared with the 2-tailed unpaired Student’s *t* test. *P* values less than 0.05 were considered significant.

### Study approval.

Animal experiments were approved by the Institutional Animal Studies Committee at Washington University. Animals received humane care in compliance with the *Guide for the Care and Use of Laboratory Animals* (National Academies Press, 2011) and with the Principles of Laboratory Animal Care formulated by the National Society for Medical Research. Human protocols were approved by the Institutional Review Board at Washington University School of Medicine (no. 201811073).

### Data availability.

RNA sequencing data generated in this study were deposited in the NCBI’s Gene Expression Omnibus (GEO) database under accession codes GSE251965 and GSE251931. The code was uploaded to https://github.com/jamrute/2023_Kreisel_Foxp3_Transplant Values for data presented in the figures are provided in the Supporting Data Values file.

## Author contributions

WL, YT, YZB, YY, HMS, JMA, AIB, ZL, MT, and JMG conducted experiments. WL, YT, YZB, AB, RGN, RRH, VP, HRT, MNA, AA, PTS, KJL, ASK, AEG, and DK contributed to study design. All authors reviewed and revised the manuscript. WL, YT, YZB, HMS, JMA, AIB, ZL, JMG, JHR, MNA, and MT analyzed and interpreted the data. WL, YT, YZB, HMS, and DK wrote the manuscript. DK designed and supervised the study. The order of the shared first authors (WL, YT, and YZB) was determined based on relative contributions to study design, experimental work, and preparation of manuscript draft and figures.

## Supplementary Material

Supplemental data

Supplemental video 1

Supplemental video 2

Supplemental video 3

Supporting data values

## Version 1 03/18/2025

In-Press Preview

## Version 2 05/15/2025

Electronic publication
