# Supplementary material for: Maintenance of graft tissue–resident Foxp3+ cells is necessary for lung transplant tolerance in mice
Source: J Clin Invest. 2025 Mar 18;135(10):e178975. doi: 10.1172/JCI178975 (PMC12077894; doi:10.1172/JCI178975)
Supplement: Supplemental data [file jci-135-178975-s289.pdf]

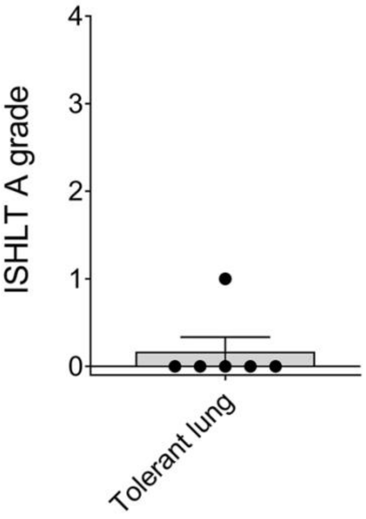

Supplemental Figure 1. ISHLT A rejection grades of Balb/c lungs ≥30 days after transplantation into CSB-treated B6 mice (n=6).

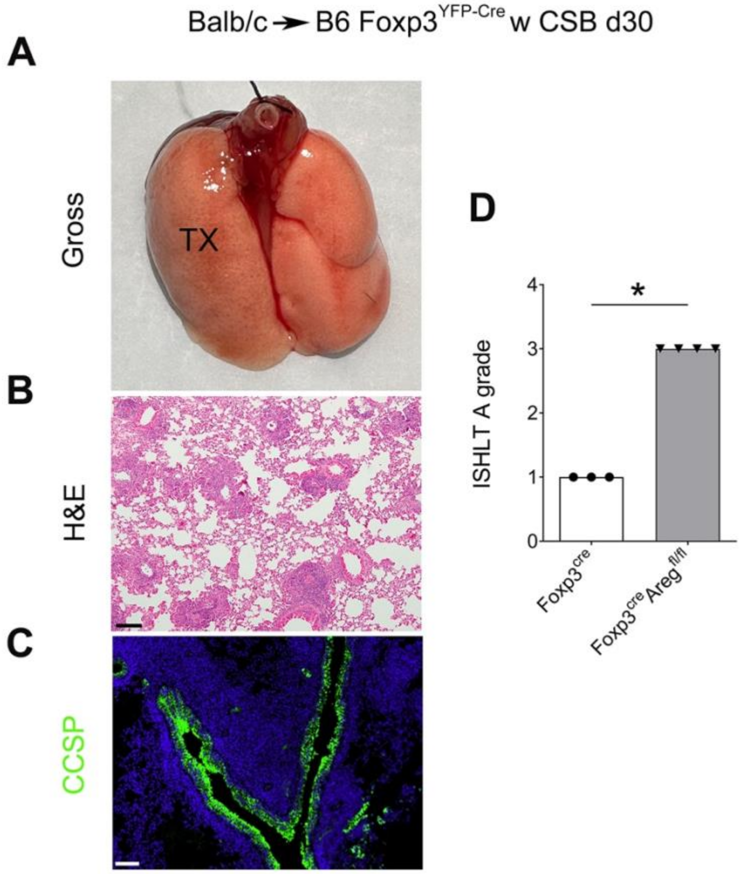

Supplemental Figure 2. Lung transplant tolerance is maintained when recipient Foxp3^{+} cells express amphiregulin. A. Gross image, **B.** H&E staining, and **C.** CCSP immunofluorescence staining of Balb/c lungs 30 days after transplantation into CSB-treated B6 Foxp3-YFP-Cre mice (n=2). **D.** ISHLT A rejection grades of Balb/c lungs 30 days after transplantation into CSB-treated B6 Foxp3-YFP-Cre and Foxp3-YFP-Cre *Areg*$^{fl/fl}$ mice (n=4). Scale bars 100 µm. (d = day, CSB = costimulatory blockade, TX = transplanted lung, H&E = hematoxylin and eosin, CCSP = club cell secretory protein)

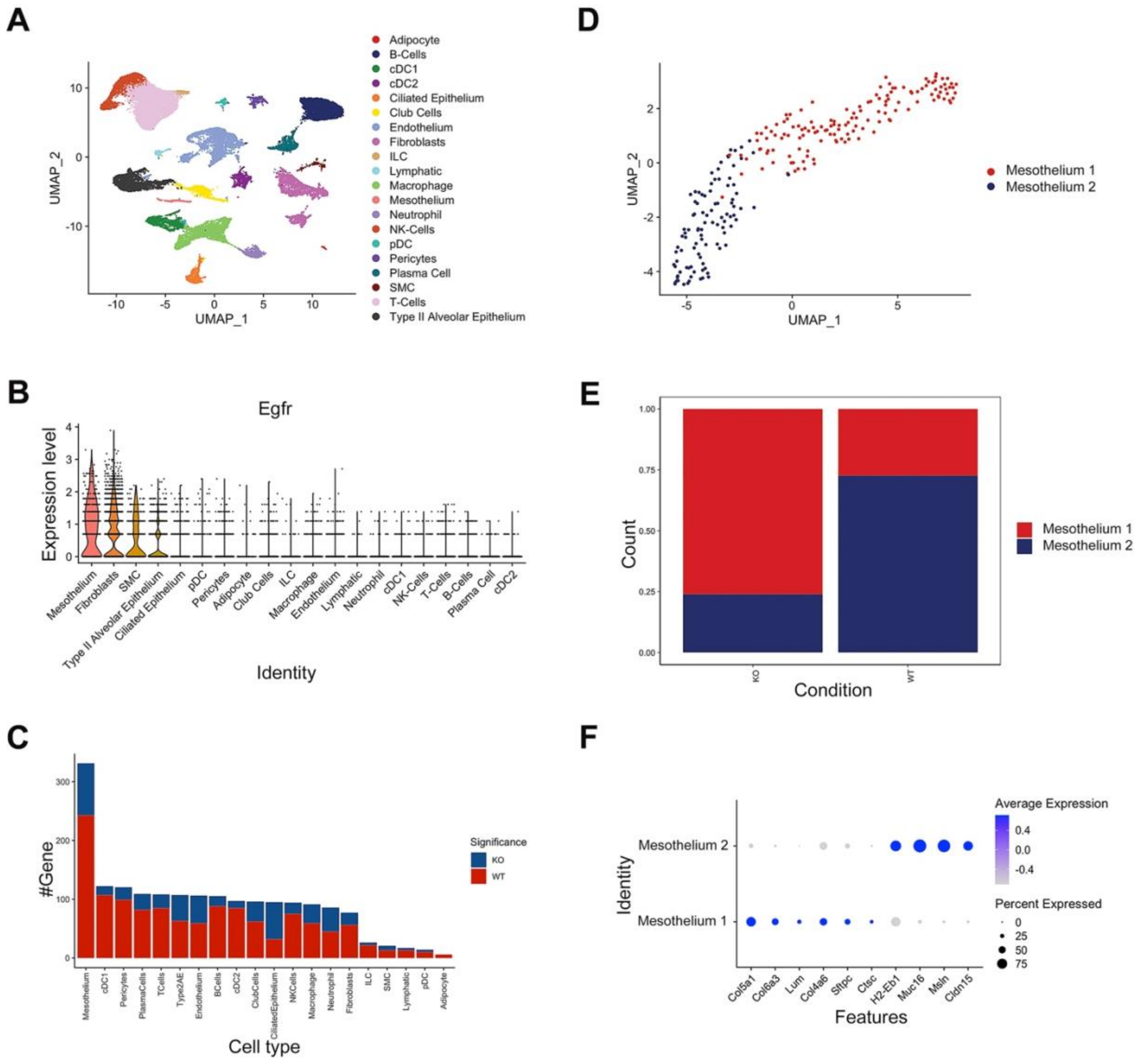

Supplemental Figure 3. Foxp3^{+} cell-derived amphiregulin induces transcriptional changes in lung allografts. Balb/c lungs were examined by single nuclear RNA sequencing 14 days after transplantation into CSB-treated B6 Foxp3-YFP-Cre *Areg*$^{fl/fl}$ or B6 Foxp3-YFP-Cre controls. Two lung allografts were pooled per group. **A**. UMAP plot colored by cell type in allografts. **B.** Violin plots showing expression of *Egfr* in stromal and immune cell populations in lung allografts. **C.** Number of differentially expressed genes in stromal and immune cell populations in lung allografts. Red: upregulated in control recipients; blue: upregulated in B6 Foxp3-YFP-Cre *Areg*$^{fl/fl}$ recipients. Statistically significant genes were used (log2FC > 0.25 and adjusted p-value < 0.05). **D.** UMAP plot of mesothelial cell states. **E.** Bar graph showing relative compositions of mesothelial cell states in experimental groups. **F.** Graph depicting differentially expressed genes between mesothelial cell states. WT: wildtype; KO: knockout; SMC: smooth muscle cell; pDC: plasmacytoid dendritic cell; ILC: innate lymphoid cell; cDC: classical dendritic cell.

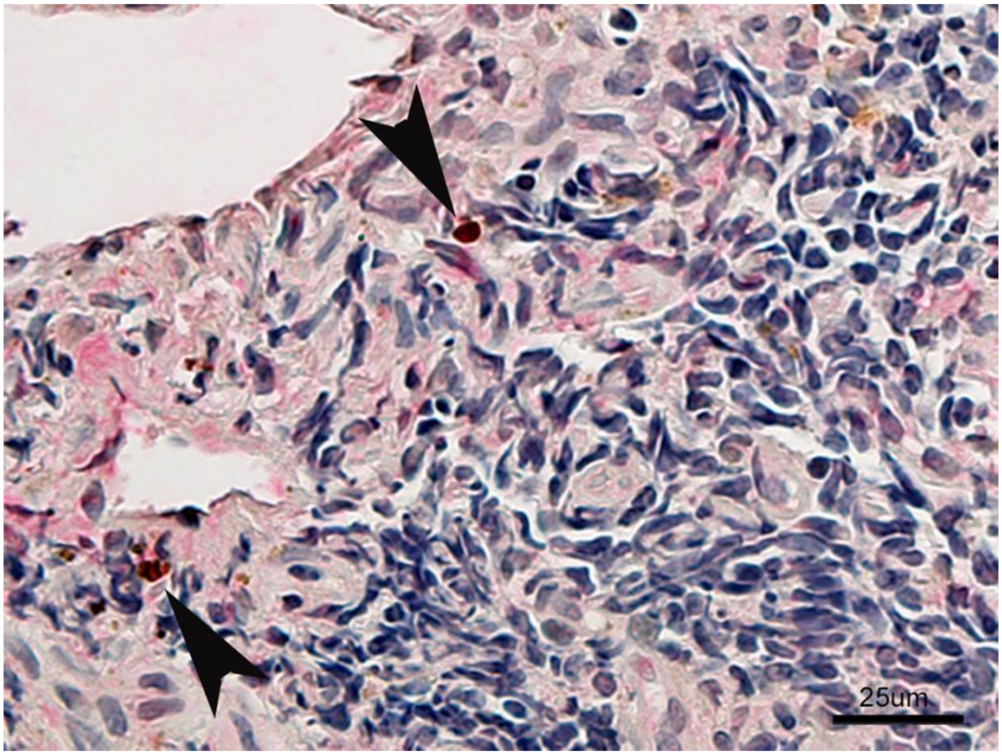

Supplemental Figure 4. Immunostaining of tissue from transbronchial biopsy of human lung transplant patient with A0 rejection and BALT shows co-localization of Foxp3 (brown) and amphiregulin (red) within BALT. Co-localization of Foxp3 and amphiregulin was observed in transbronchial biopsies from 4/5 patients with A0 rejection and presence of BALT. Arrows point to co-localization of Foxp3 and amphiregulin staining. Scale bar 25μm.

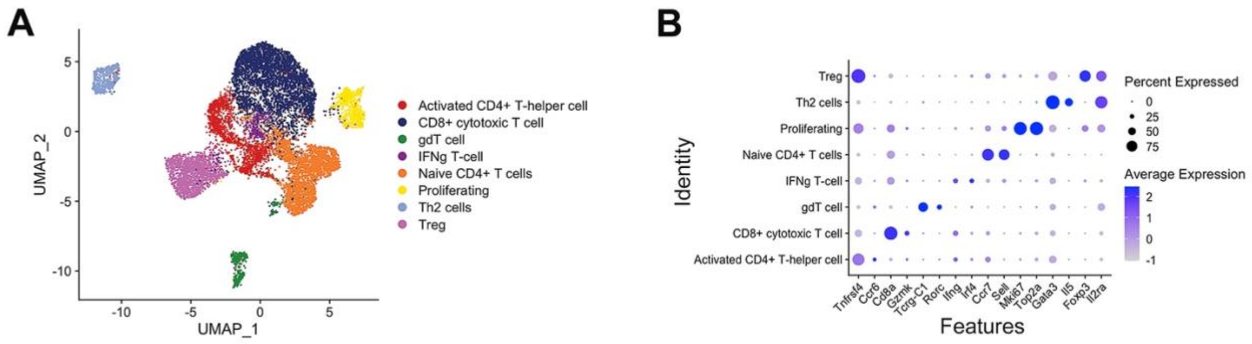

Supplemental Figure 5. Several T cell populations reside in tolerant lung allografts. A. UMAP plot showing 8 T cell populations and **B**. graph depicting differentially expressed genes between the T cell populations. Balb/c (CD45.2) lungs were transplanted into CSB-treated B6 (CD45.2) recipients and at least 30 days later re-transplanted into non-immunosuppressed B6 (CD45.1) mice. Seven and 21 days after re-transplantation, graft-resident (CD45.2) (7 days) and extravasated graft-infiltrating (CD45.1) (7 and 21 days) T cells were sorted from the lung allografts (samples were collected from 4 re-transplant recipients and pooled) and processed for single cell RNA sequencing. gdT cell: γδ T cell; Treg: regulatory T cell

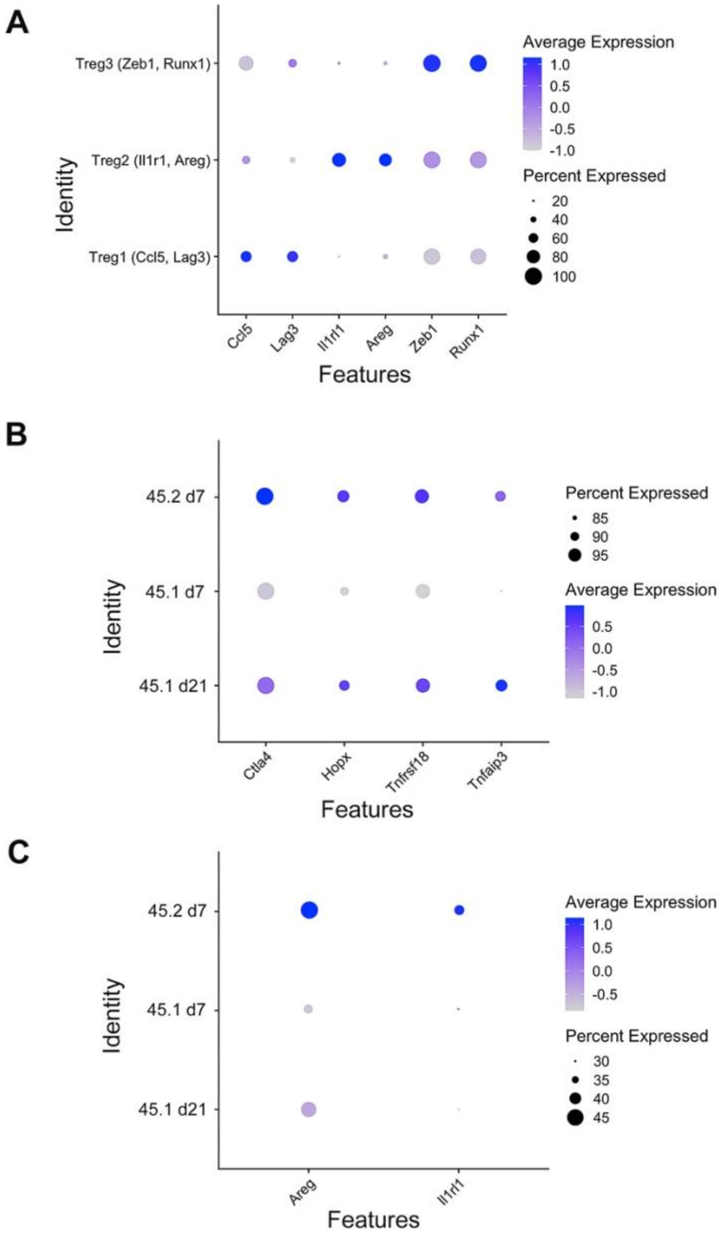

Supplemental Figure 6. Graft-infiltrating regulatory T cells acquire a transcriptional profile resembling that of graft-resident regulatory T cells over time. A. Graph depicting differentially expressed genes between regulatory T cell populations (UMAP shown in Figure 3C). **B.** and **C.** Graph depicting differentially expressed genes between graft-resident (CD45.2) (7 days) and extravasated graft-infiltrating (CD45.1) (7 and 21 days) regulatory T cells in tolerant Balb/c (CD45.2) lung allografts, initially transplanted for ≥30 days into CSB-treated B6 CD45.2 mice and then re-transplanted into secondary non-immunosuppressed B6 CD45.1 recipients. (4 pooled lung allografts per time point)

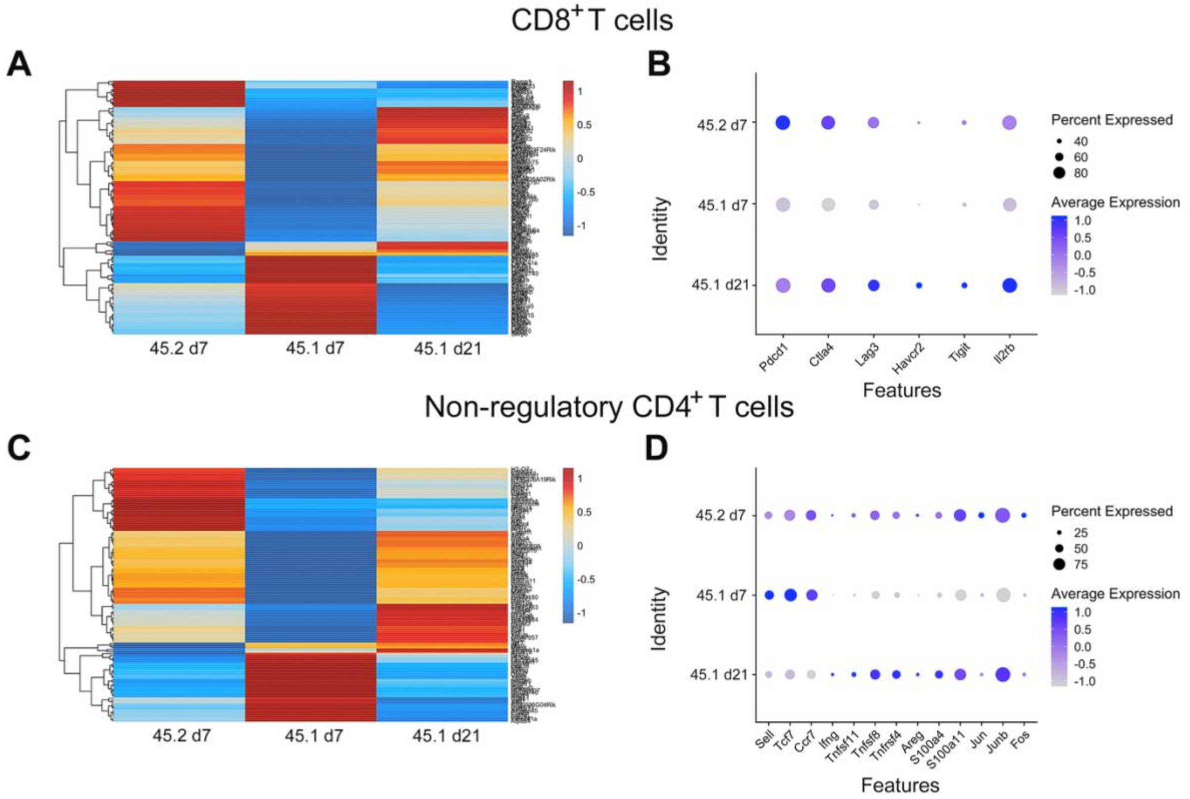

Supplemental Figure 7. Graft-infiltrating CD8^{+} and non-regulatory CD4^{+} T cells acquire a transcriptional profile resembling that of graft-resident regulatory T cells over time. Heatmaps of statistically significant (log2FC > 0.25, adjusted p-value < 0.05) differentially expressed genes and graphs representing select genes between extravasated graft-infiltrating CD45.1 (days 7 and 21) and graft-resident CD45.2 (day 7) CD8^{+} (**A, B**) and non-regulatory CD4^{+} T cells (**C**, **D**) in tolerant Balb/c (CD45.2) lung allografts (initially transplanted for ≥30 days into CSB-treated B6 CD45.2 mice) after re-transplantation into secondary non-immunosuppressed B6 CD45.1 recipients. (4 pooled lung allografts per time point).

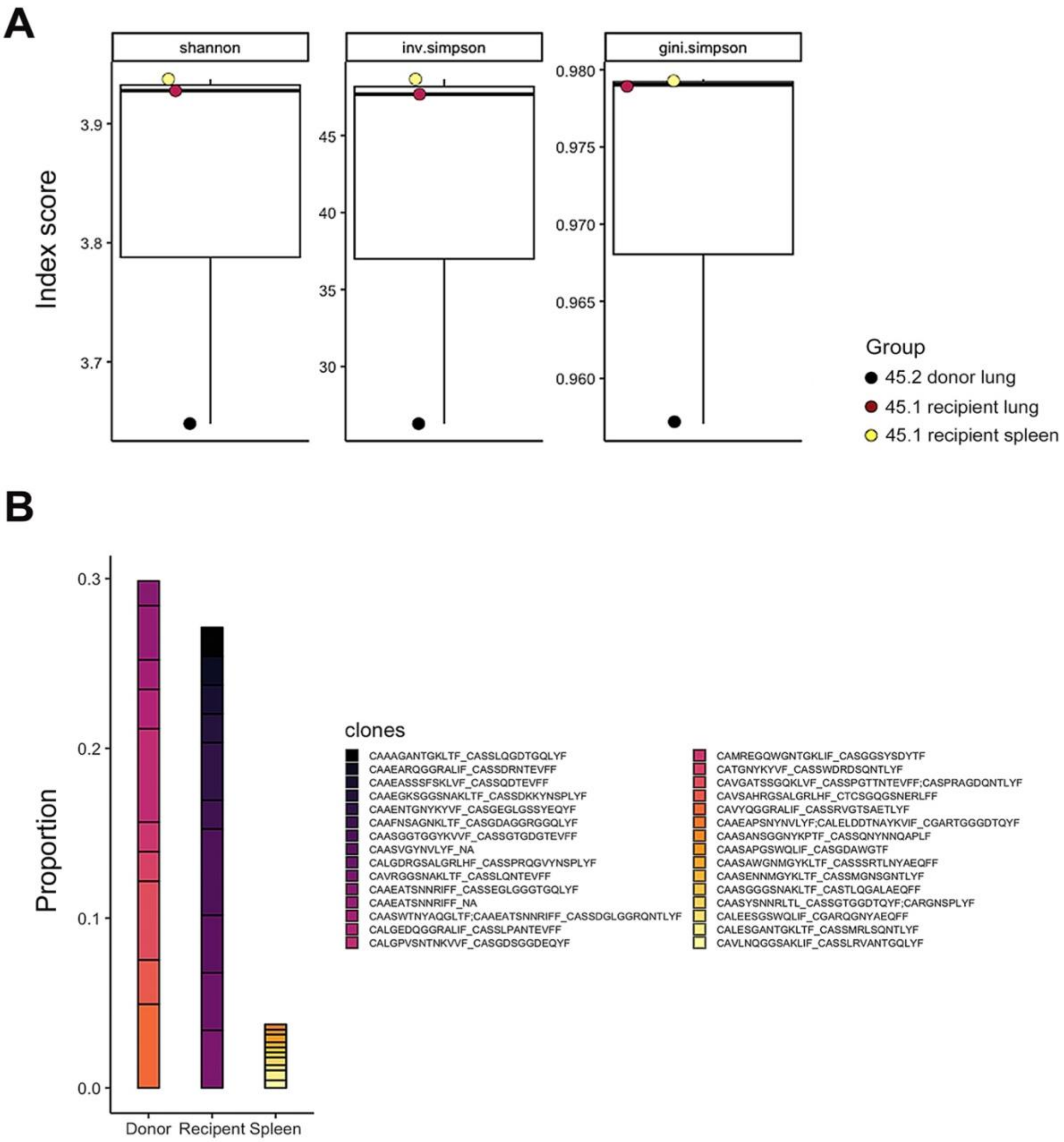

Supplemental Figure 8. Newly graft-infiltrating and splenic regulatory T cells display a higher degree of TCR clonal diversity than graft-resident regulatory T cells. A. Shannon, inverse Simpson and Gini-Simpson coefficient indices of clonal expansion between graft-resident (CD45.2) and graft-infiltrating (CD45.1) regulatory T cells in tolerant Balb/c (CD45.2) lung allografts as well as splenic regulatory T cells (initially transplanted for ≥30 days into CSB-treated B6 CD45.2 mice) 4 days after re-transplantation into secondary non-immunosuppressed B6 CD45.1 recipients. **B.** Proportion of TCR clones grouped by condition (4 pooled lung allografts and spleens)

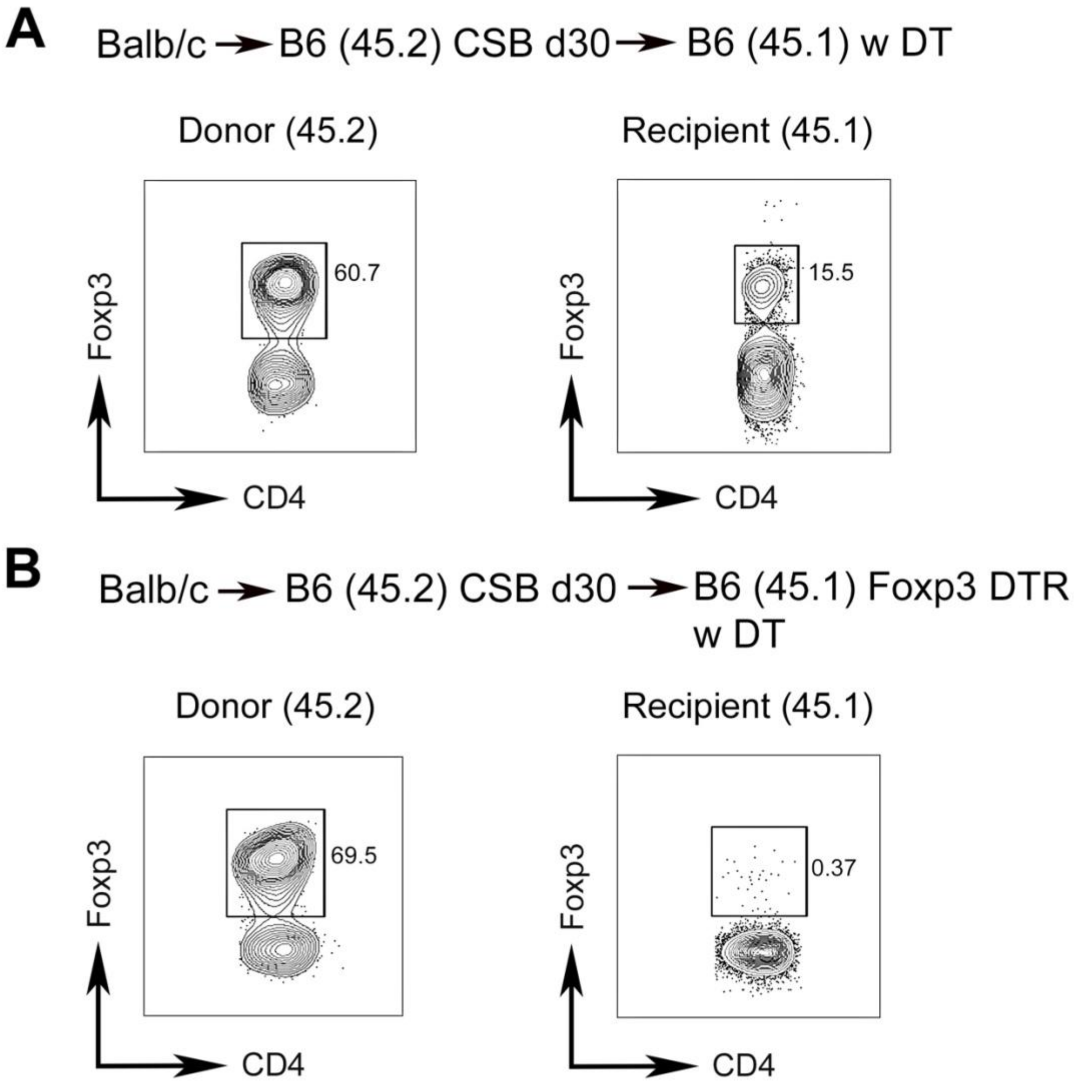

Supplemental Figure 9. Foxp3$^+$ cells in re-transplant recipients of tolerant lung allografts are depleted after administration of diphtheria toxin. Balb (CD45.2) lungs were transplanted into CSB-treated B6 (CD45.2) recipients and at least 30 days later re-transplanted into diphtheria toxin-treated non-immunosuppressed **A.** B6 CD45.1 or **B.** B6 Foxp3-DTR CD45.1 mice. Representative flow cytometric plots of graft-resident (donor; CD45.2$^+$CD45.1$^-$) versus graft-infiltrating live (recipient; CD45.2$^-$CD45.1$^+$) CD90$^+$CD4$^+$CD8$^-$Foxp3$^+$ cells seven days after re-transplantation (n≥3). (CSB = costimulatory blockade, d = day, DT = diphtheria toxin, DTR = diphtheria toxin receptor)

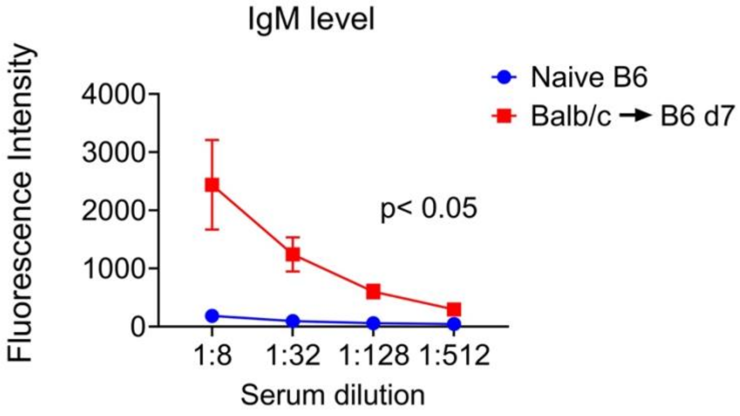

Supplemental Figure 10. Flow cytometric analysis of serum IgM DSA (anti-Balb/c) titers (expressed as mean fluorescence intensity; 1:8 dilution) in naïve B6 mice as well as 7 days after transplantation of Balb/c lungs into non-immunosuppressed B6 mice (n=4)**.**

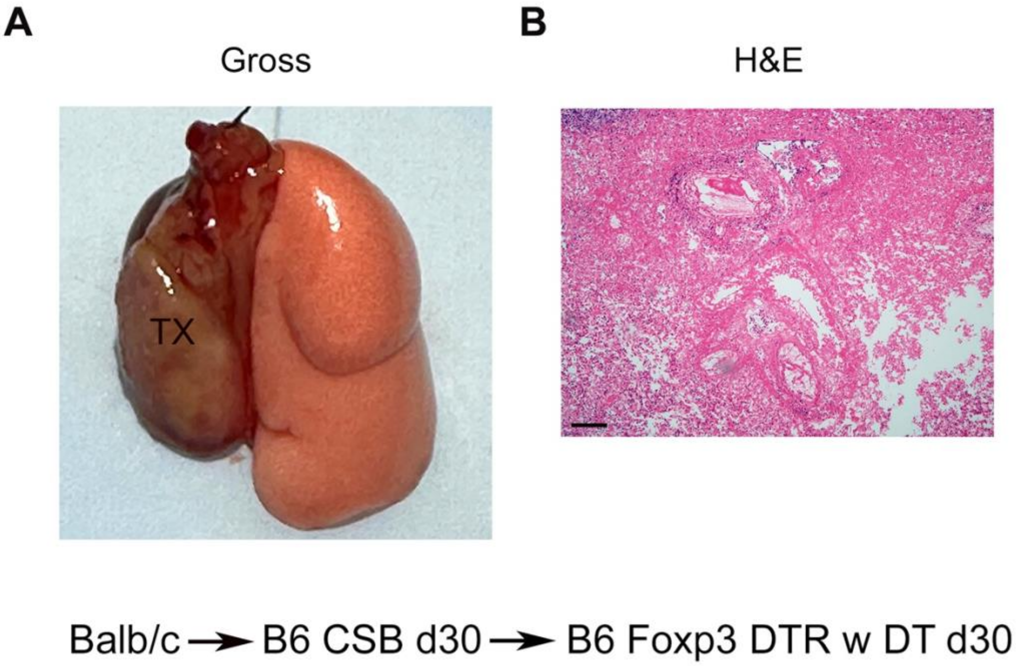

Supplemental Figure 11. Re-transplanted lung allografts are rejected after depletion of recipient Foxp3$^+$ cells. A. Gross (left) and **B.** histological appearance (H&E) (right) of left lung from Balb/c donor initially transplanted into CSB-treated B6 primary recipient and then ≥30 days later re-transplanted into non-immunosuppressed DT-treated B6 Foxp3-DTR secondary recipient. Grafts were examined 30 days after re-transplantation (n=4). Scale bar 100 μm. (d = day, CSB = costimulatory blockade, TX = transplanted lung, H&E = hematoxylin and eosin)

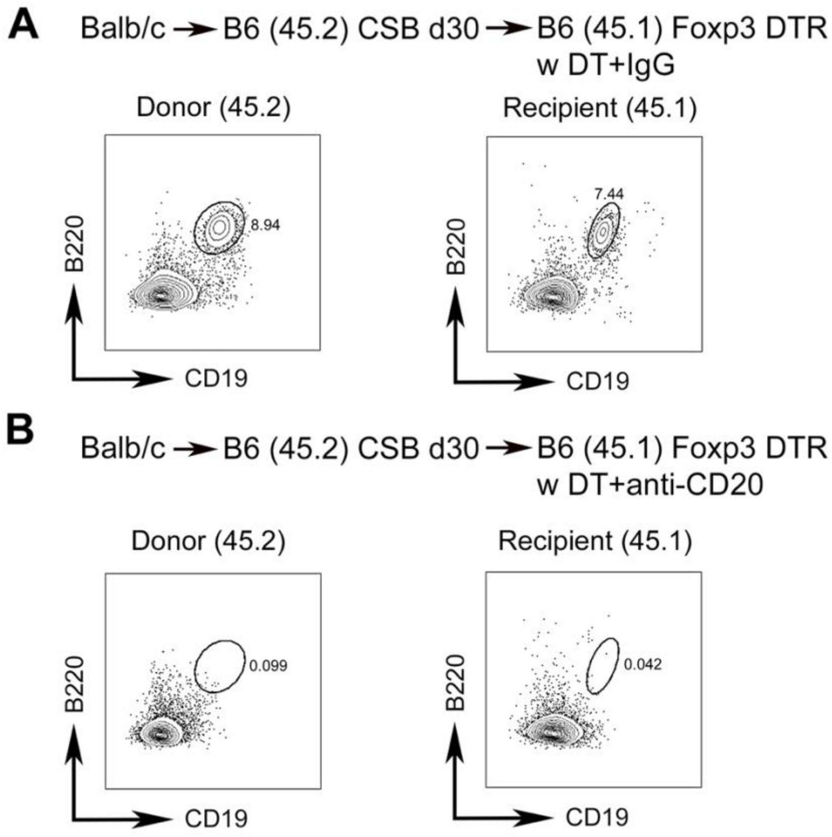

Supplemental Figure 12. Intragraft B cells are depleted following administration of anti-CD20 antibodies to re-transplant recipients. Balb (CD45.2) lungs were transplanted into CSB-treated B6 (CD45.2) recipients and at least 30 days later re-transplanted into DT-treated non-immunosuppressed B6 Foxp3-DTR CD45.1 mice that received **A.** control IgG or **B.** anti-CD20 antibodies. Representative flow cytometric plots of graft-resident (donor; $CD45.2^{+}CD45.1^{-}$) versus graft-infiltrating live (recipient; $CD45.2^{-}CD45.1^{+}$) $B220^{+}CD19^{+}$ B cells seven days after re-transplantation (n=4). (CSB = costimulatory blockade, d = day, DT = diphtheria toxin, DTR = diphtheria toxin receptor)

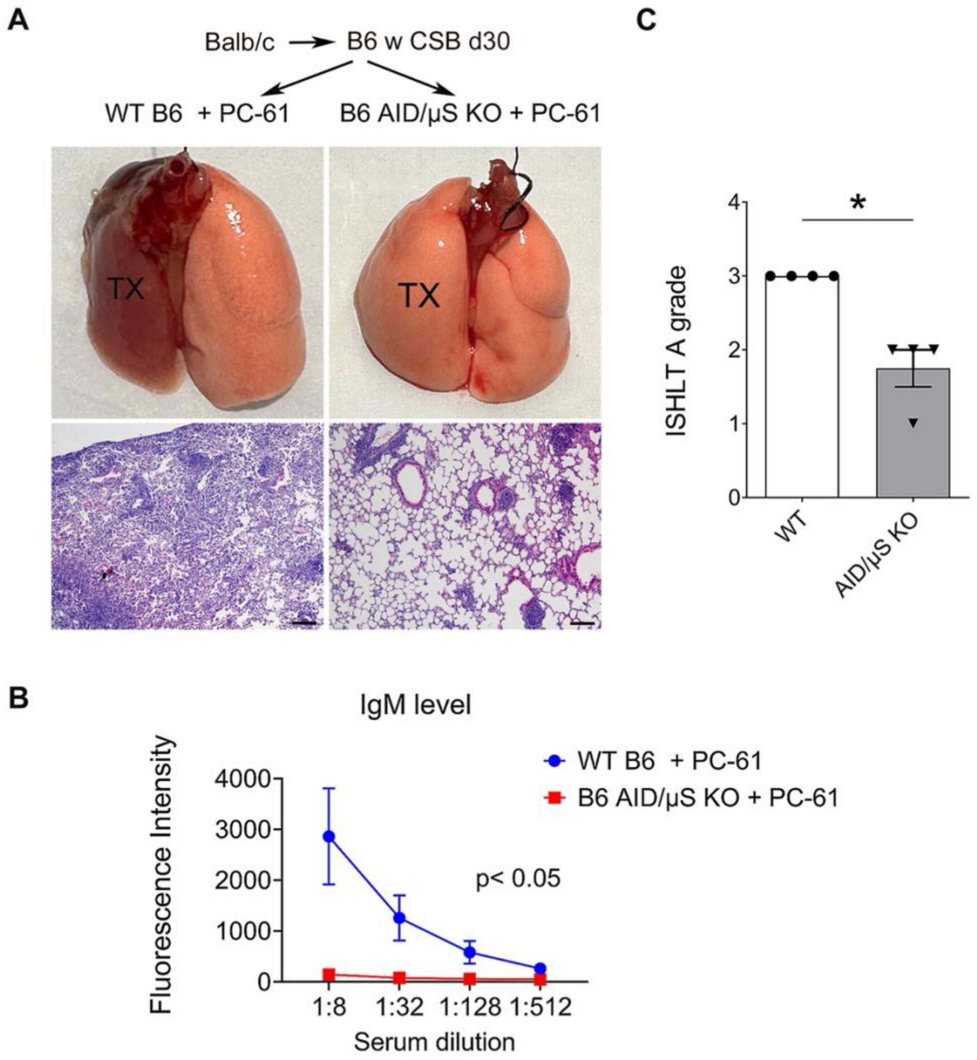

Supplemental Figure 13. Lung transplant rejection after anti-CD25 antibody treatment is attenuated when recipient B cells cannot produce alloantibodies. A. Gross (top) and histological (H&E) (bottom) images of Balb/c lungs that were initially transplanted into CSB-treated B6 mice and then at least 30 days later re-transplanted into anti-CD25 antibody (PC61)-treated non-immunosuppressed B6 wildtype (left) or AID/µS-knockout (right) recipients (n=4). Scale bar represents 100 µm. **B.** Flow cytometric analysis of serum IgM DSA titers (expressed as mean fluorescence intensity) and **C.** ISHLT A rejection grades in re-transplant recipients described in **A** (n=4). KO=knockout, *<0.05.

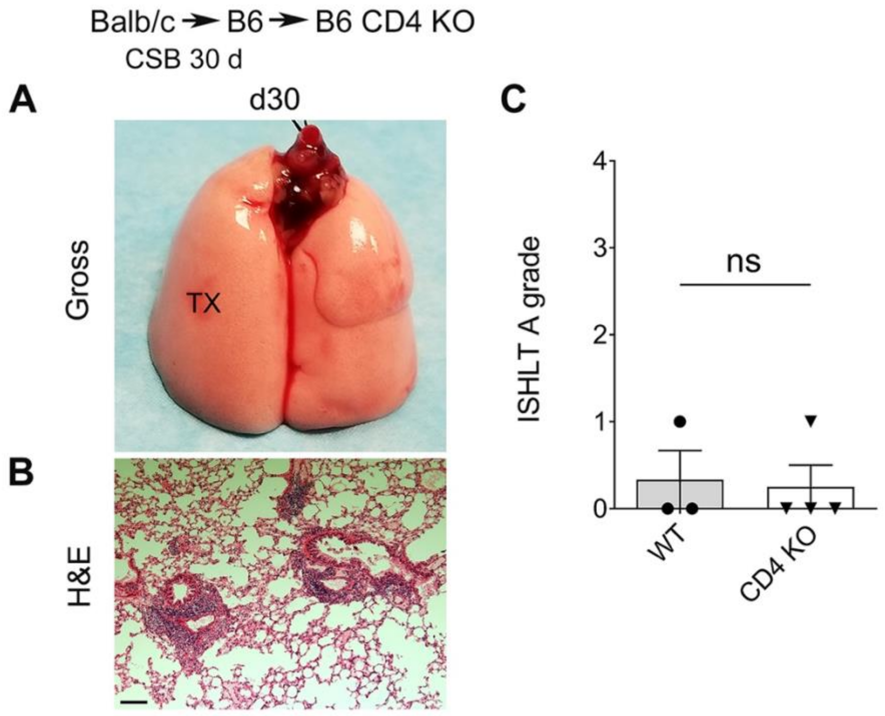

Supplemental Figure 14. Lung allograft tolerance is maintained when re-transplant recipients lack CD4 cells. A. Gross and **B.** histological (H&E) images and **C**. ISHLT A rejection grades of Balb/c lungs that were initially transplanted into CSB-treated B6 mice and then at least 30 days later re-transplanted into non-immunosuppressed B6 CD4 knockout recipients. Grafts were examined 30 days after re-transplantation (n≥3). Scale bar 100 µm. (CSB = costimulatory blockade, d = day, KO = knockout, Tx = transplanted lung, H&E = hematoxylin and eosin)

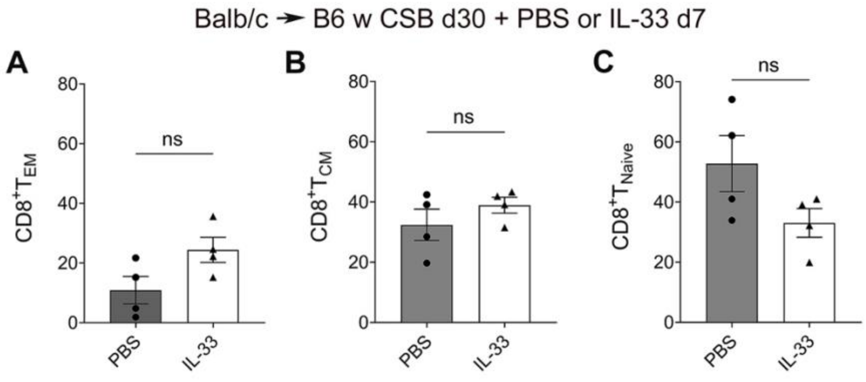

Supplemental Figure 15. IL-33 administration does not result in changes of $CD8^+$ T cell memory phenotype in tolerant lung allografts. Balb/c lungs were transplanted into CSB-treated B6 recipients. At least 30 days after transplantation, recipients were treated with IL-33 or PBS and grafts were analyzed 7 days later. Representative flow cytometry plots and quantification of abundance of **A.** effector memory ($CD44^{hi}CD62L^{lo}$), **B.** central memory ($CD44^{hi}CD62L^{hi}$), and **C.** naïve ($CD44^{lo}CD62L^{hi}$) $CD45^+CD90.2^+CD4^-CD8^+$T cells (n=4). Results expressed as mean ± SEM. (CSB = costimulatory blockade, d = day, PBS = phosphate-buffered saline, T_{EM} = T effector memory, T_{CM} = T central memory, ns = not significant)

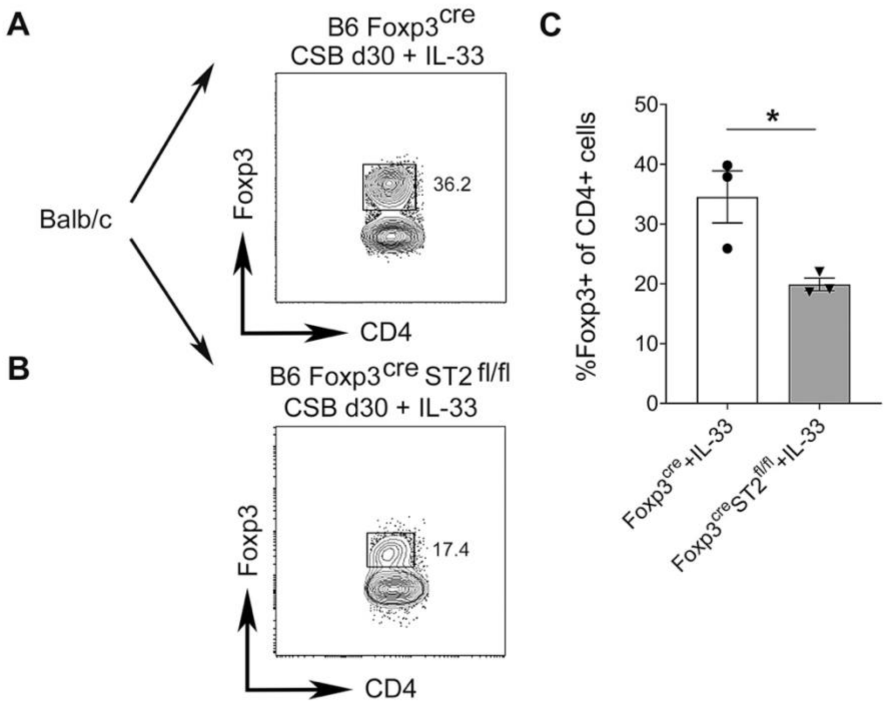

Supplemental Figure 16. Expansion of Foxp3⁺ cells in lung allografts after local IL-33 administration is dependent on *St2* expression by Foxp3⁺ cells. Balb/c lungs were transplanted into CSB-treated B6 Foxp3-YFP-Cre or B6 Foxp3-YFP-Cre *St2*$^{fl/fl}$ recipients. At least 30 days after transplantation, recipients were treated with IL-33 or PBS and grafts were analyzed 7 days later. Contour plots depicting percentage of Foxp3-expressing intragraft $CD45^+CD90.2^+CD4^+CD8^-$ T cells after transplantation into CSB-treated **A**. B6 Foxp3-YFP-Cre and **B.** B6 Foxp3-YFP-Cre *St2*$^{fl/fl}$ recipients (n=3). The comparative analysis between the two groups is depicted in **C**. *<0.05

Supplemental Video 1. Graft-infiltrating recipient Foxp3$^+$ cells interact with CD11c$^+$ cells in tolerant lung allografts. Balb/c lungs, initially transplanted into CSB-treated B6 CD11c-EYFP mice and then at least 30 days later re-transplanted into non-immunosuppressed B6 Foxp3-IRES-GFP hosts were imaged with intravital two-photon microscopy three days after re-transplantation (n=3). CD11c$^+$ cells within BALT are green and graft-infiltrating Foxp3$^+$ cells are blue. Scale bar 20 μm. Circle depicts relevant cellular interactions. (GFP = green fluorescent protein, YFP = yellow fluorescent protein; rhodamine dextran labeling vessels red)

Supplemental Video 2. Graft-infiltrating recipient Foxp3$^+$ cells interact with graft-resident Foxp3$^+$ cells in tolerant lung allografts. Balb/c lungs, initially transplanted into CSB-treated B6 Foxp3-IRES-GFP mice and then at least 30 days later re-transplanted into non-immunosuppressed B6.Foxp3-IRES-RFP recipients were imaged with intravital two-photon microscopy three days after re-transplantation (n=3). Graft-resident Foxp3$^+$ cells are green and graft-infiltrating Foxp3$^+$ cells are red. Scale bar 20 μm. Circle depicts relevant cellular interactions. (GFP = green fluorescent protein, RFP = red fluorescent protein)

Supplemental Video 3. Graft-infiltrating recipient Foxp3$^+$ cells interact with graft-infiltrating B cells in tolerant lung allografts. Balb/c lungs were initially transplanted into CSB-treated B6 CD11c-EYFP mice and then re-transplanted into non-immunosuppressed B6 Foxp3-IRES-GFP hosts at least 30 days later. Recipient-matched B6 CMTMR-labeled B cells were injected into recipients two days after re-transplantation and allografts were imaged with intravital two-photon microscopy the following day (n=4). CD11c$^+$ cells within BALT are yellow, graft-infiltrating Foxp3$^+$ cells are green and graft-infiltrating B cells are red. Scale bar 20 μm. Circle depicts relevant cellular interactions. (YFP = yellow fluorescent protein, GFP = green fluorescent protein, CMTMR = rhodamine-based red cell dye)
